# Global meta-analysis of plasma-activated water for improving crop establishment, productivity, and health

**DOI:** 10.3389/fpls.2026.1754073

**Published:** 2026-04-15

**Authors:** Nawab Ali, Younsuk Dong

**Affiliations:** Biosystems and Agricultural Engineering, Michigan State University, East Lansing, MI, United States

**Keywords:** biophysicochemical traits, disease control, plasma activated water (PAW), productivity, reactive species, sustainable agriculture

## Abstract

Plasma-activated water (PAW) has emerged as a promising eco-friendly technology and a sustainable alternative to chemical fertilizers in agriculture due to its role in plant growth, nutrition, and disease suppression. To comprehensively evaluate its effectiveness, a meta-analysis was conducted using published studies comprising diverse crops, experimental conditions, and PAW generation chemistry. Log response ratios (LnRR) and pooled estimates with 95% confidence intervals were calculated, and forest plots were generated for each response variable. Results revealed that PAW application significantly improved plant growth and pigment content (chlorophyll a, b, and c and carotenoids), along with total soluble solids (TSS), total soluble protein (TSP), ascorbate peroxidase (APX), catalase (CAT), superoxidase dismutase (SOD), and total phenolic content (TPC) supporting improved metabolic activity. Moreover, a significant reduction in disease incidence and severity under PAW application ensured enhanced plant defense responses. Plasma activation altered water chemistry by lowering pH and increasing electrical conductivity, nitrate, nitrite, H_2_O_2_, and oxidation–reduction potential (ORP), reflecting the stable presence of reactive oxygen and nitrogen species (RONS). This comprehensive meta-analysis indicates that PAW consistently improves plant growth, biochemical characteristics, yield, and disease resistance. The acidifying nature of PAW also influences soil microbial dynamics, potentially enhancing nutrient cycling and long-term soil fertility. Collectively, the findings support PAW application as a multifunctional strategy for advancing sustainable agriculture through improved plant performance. The integration of solar-powered irrigation systems with onsite PAW activation and water quality sensors demonstrates a scalable pathway for precise, resilient irrigation and nutrient delivery in agriculture.

## 1 Introduction

Water supplies are deteriorating due to climate change, land use changes, agricultural and urban expansion, and overexploitation (Scanlon et al., 2023). Along with these pressures, soil degradation and agrochemical application are the most prominent challenges faced by global agriculture (Yasir et al., 2025). The modern crop production system necessitates irrigation strategies that not only enhance water use efficiency but also favor nutrient management and promote plant health (Mubeen et al., 2025). Plasma, often referred to as the fourth state of matter, consists of high-energy particles such as ions, electrons, excited atoms and molecules, and free radicals (Guo et al., 2021). Besides these chemical components, the cold plasma generation involves certain physical factors that consisted of ultraviolet (UV) radiation, electromagnetic emissions, and transient electric fields acting independently or synergistically with reactive oxygen and nitrogen species (RONS) (Obradović et al., 2008; Kaneko et al., 2015; Robert et al., 2015, Robert et al., 2025; Chung et al., 2020; Vijayarangan et al., 2026). These components exhibit unique chemical reactivity, including strong oxidative potential and the ability to degrade organic compounds (Guo et al., 2021). When plasma interacts with gases, it generates reactive species that can dissolve into liquids, forming plasma-activated liquids (PAL) or plasma-activated water (PAW) (Puač and Škoro, 2025). These modified liquids possess enhanced physicochemical properties that are beneficial for applications ranging from microbial decontamination to plant growth stimulation (Guo et al., 2021). Plasma is classified as high-temperature plasma (HTP), typically seen in nuclear fusion, and low-temperature plasma (LTP), which includes thermal plasma (TP) and cold plasma (CP). The TP forms at temperatures above 20,000 K, while CP operates at 30–60 °C and is generated using methods like dielectric barrier discharge (DBD), plasma jets, and corona discharges (Waghmare, 2021; Liu et al., 2022; Rathore and Nema, 2025). CP is a sustainable, low-energy technology that enhances crop productivity under stress with minimal environmental impact, offering a safer alternative to conventional agrochemicals (Fernandes and Rodrigues, 2025). HTP exists in thermodynamic equilibrium at temperatures of several thousand kelvin and is primarily employed in industrial applications while LTP is a non-equilibrium plasma that generates reactive species while maintaining near-ambient bulk gas temperatures, enabling its application in PAW production, seed priming, and crop protection (Thirumdas et al., 2018).

PAW is an emerging and sustainable technology with broad applicability across the biological sciences. Its unique physicochemical properties have enabled its use in diverse fields such as plasma medicine, oncology, seed germination, plant growth promotion, sustainable agriculture, microbial decontamination, food processing, storage, and preservation (Mumtaz et al., 2023; Bhabani et al., 2024; Guo et al., 2024). PAW is produced when plasma discharges interact with water and is characterized by the presence of RONS such as hydrogen peroxide (H_2_O_2_), nitrate (NO_3_^-^), nitrite (NO_2_^-^), hydroxyl radicals (OH), and superoxide (O_2_^-^). These species impart PAW with unique physicochemical properties, including lowered pH, elevated electrical conductivity (EC), and increased oxidation–reduction potential (ORP) (Fernandes and Rodrigues, 2025). Collectively, these properties underpin PAW’s antimicrobial effects, ability to modify nutrient dynamics, and potential to stimulate plant physiological and biochemical responses. Laboratory and greenhouse studies have consistently reported enhanced seed germination, improved root and shoot biomass, increased pigment accumulation, and reduced disease incidence in crops treated with PAW (Guo et al., 2021). PAW has shown effectiveness in enhancing seed germination, disinfection, and plant growth (Graves et al., 2019). Direct seed treatment with PAW alters the seed coat, improving germination (Veerana et al., 2024). Factors like plasma dosage, duration, gas type, and moisture influence seedling development (Adhikari et al., 2020). Short treatments improve biomass, while prolonged exposure may inhibit growth (Šerá et al., 2021; Jirešová et al., 2022; Scholtz et al., 2025). PAW benefits reproductive traits in crops like peanuts (Song et al., 2023), soybeans (Lo Porto et al., 2018), tomatoes (Pérez-Pizá et al., 2019), and okra (Kumar et al., 2018) and increases pod number and grain weight in oilseed rape (Li et al., 2025). It also boosts plant immunity (Panngom et al., 2014), supports post-harvest disinfection (Siddique et al., 2018), and aids soil treatment (Fumiaki et al, 2016). CP stimulates growth regulators like auxins and gibberellins, enhancing stress resistance (Fernandes and Rodrigues, 2025).

Despite these promising findings, the application of PAW in large-scale irrigation remains limited. The strong acidification and variability in reactive species concentrations pose challenges for sensitive crops and soils, while short-lived radicals constrain storage and transport (Andrade et al., 2025). Moreover, most evidence to date has been generated under controlled experimental conditions, with relatively little validation in field settings or across diverse environments. Addressing these limitations requires standardized PAW generation protocols and the development of scalable delivery systems that integrate renewable energy, water quality monitoring, and precision control, while ensuring cost-effectiveness.

Meta-analytical approaches are particularly valuable for synthesizing findings across individual research studies, enabling robust conclusions about the agronomic impacts of PAW. By pooling evidence from multiple crops, environments, and experimental designs, meta-analysis provides quantitative insights into the extent to which PAW influences germination, growth, biochemical parameters, and disease resistance. In addition, conceptual frameworks for field deployment such as solar-powered irrigation systems with onsite PAW activation chambers, real-time water quality monitoring sensors, and controlled distribution networks offer pathways for translating laboratory efficacy into real-world onsite irrigation fields. Therefore, this meta-analysis was performed to assess the PAW effect on plant growth, biochemical traits, productivity, and health, and to outline future directions for scaling up PAW into an irrigation system that integrates plasma activation with solar energy for sustainable water and nutrient management in agriculture.

## 2 Materials and methods

### 2.1 Literature search strategy and data collection

A systematic and comprehensive literature search was conducted to identify studies reporting the effect of PAW on plant establishment, growth, yield, biochemical characteristics, disease incidence, disease severity of plants, and water chemical properties. The search strategy was designed to ensure methodological rigor and relevance by applying predefined inclusion criteria. Peer-reviewed articles were retrieved from major electronic databases, including Web of Science, Scopus, Google Scholar and ScienceDirect, with the search limited to publications available up to December 2024.

**Figure 1:**
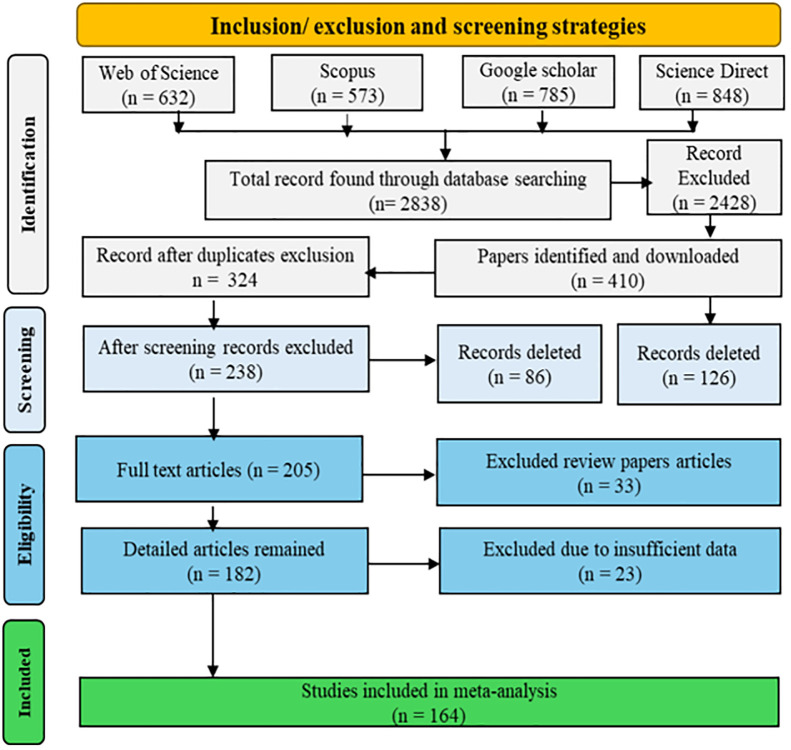
Flow diagram as per PRISMA guidelines for paper selection from Web of Science, Scopus, Google Scholar, and ScienceDirect indicating identification, screening, eligibility, and inclusion in meta-analysis.

The keywords used for literature search were “Plasma Activated Water” AND “irrigation” AND (“nutrient use efficiency” OR “nutrient uptake”) AND (“disease management” OR “pathogen control” OR “plant defense”) AND (“plant growth” OR yield OR quality) for Google Scholar, Scopus (TITLE-ABS-KEY), and “topic search” for Web of Science and ScienceDirect. The total number of searched papers through Google Scholar, Scopus, Web of Science, and ScienceDirect were 785, 573, 632, and 848, respectively. Duplicates, irrelevant papers, reviews, and papers containing no data were excluded from the searched papers as shown in the PRISMA flowchart (**Figure 1**).

#### 2.1.1 Inclusion criteria

**Figure 2:**
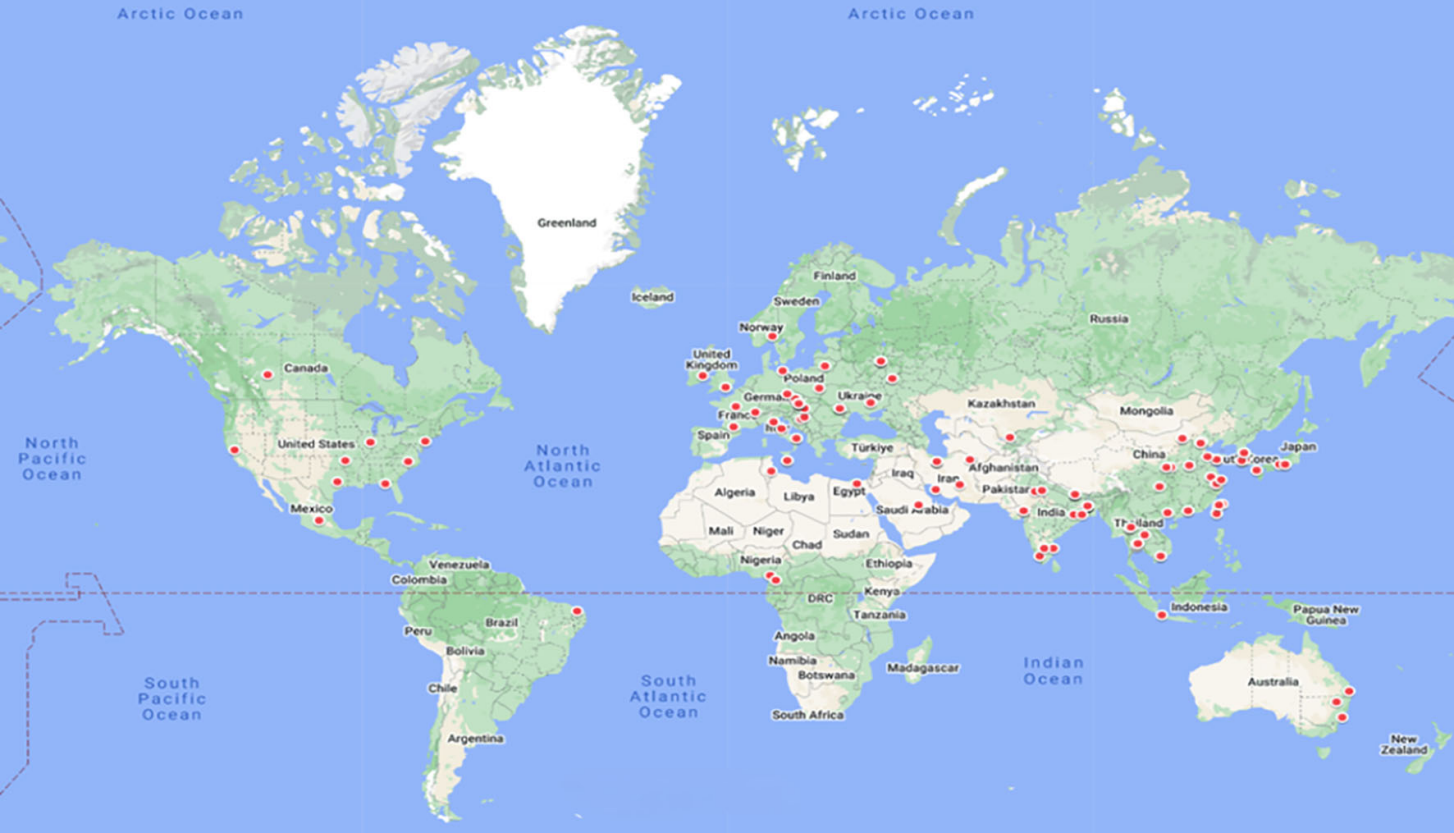
Global distribution of experimental sites that conducted research on PAW for irrigation, highlighting spatial representation of experiments included in the meta-analysis.

**Figure 3:**
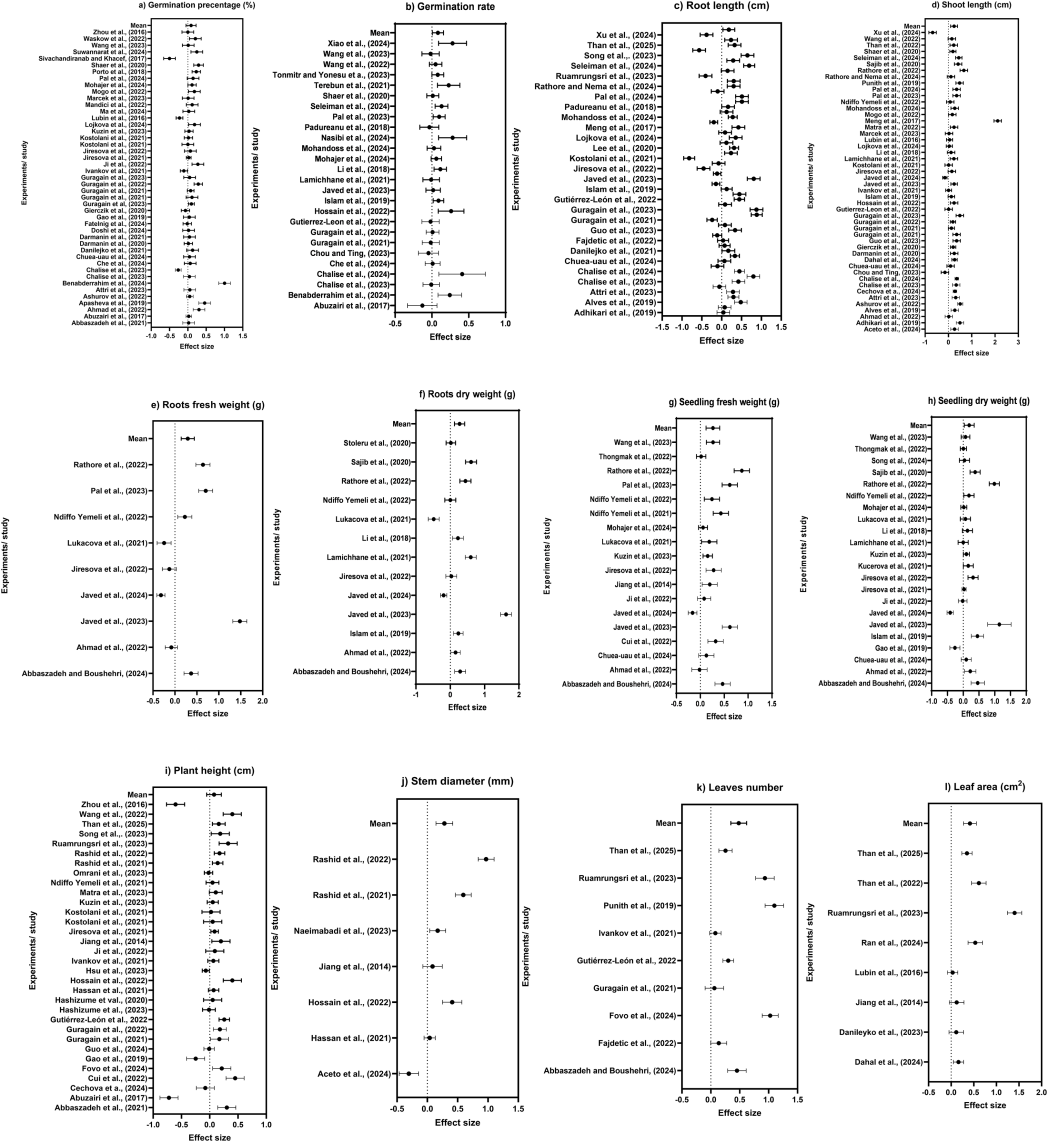
**(a)** Germination percentage, **(b)** germination rate, **(c)** root length, **(d)** shoot length, **(e)** root fresh weight, **(f)** root dry weight, **(g)** seedling fresh weight, **(h)** seedling dry weight, **(i)** plant height, **(j)** stem diameter, **(k)** leaf number, and **(l)** leaf area effect sizes expressed as log response ratios (LnRR) with 95% confidence interval comparing PAW application effects relative to control treatment.

**Figure 4:**
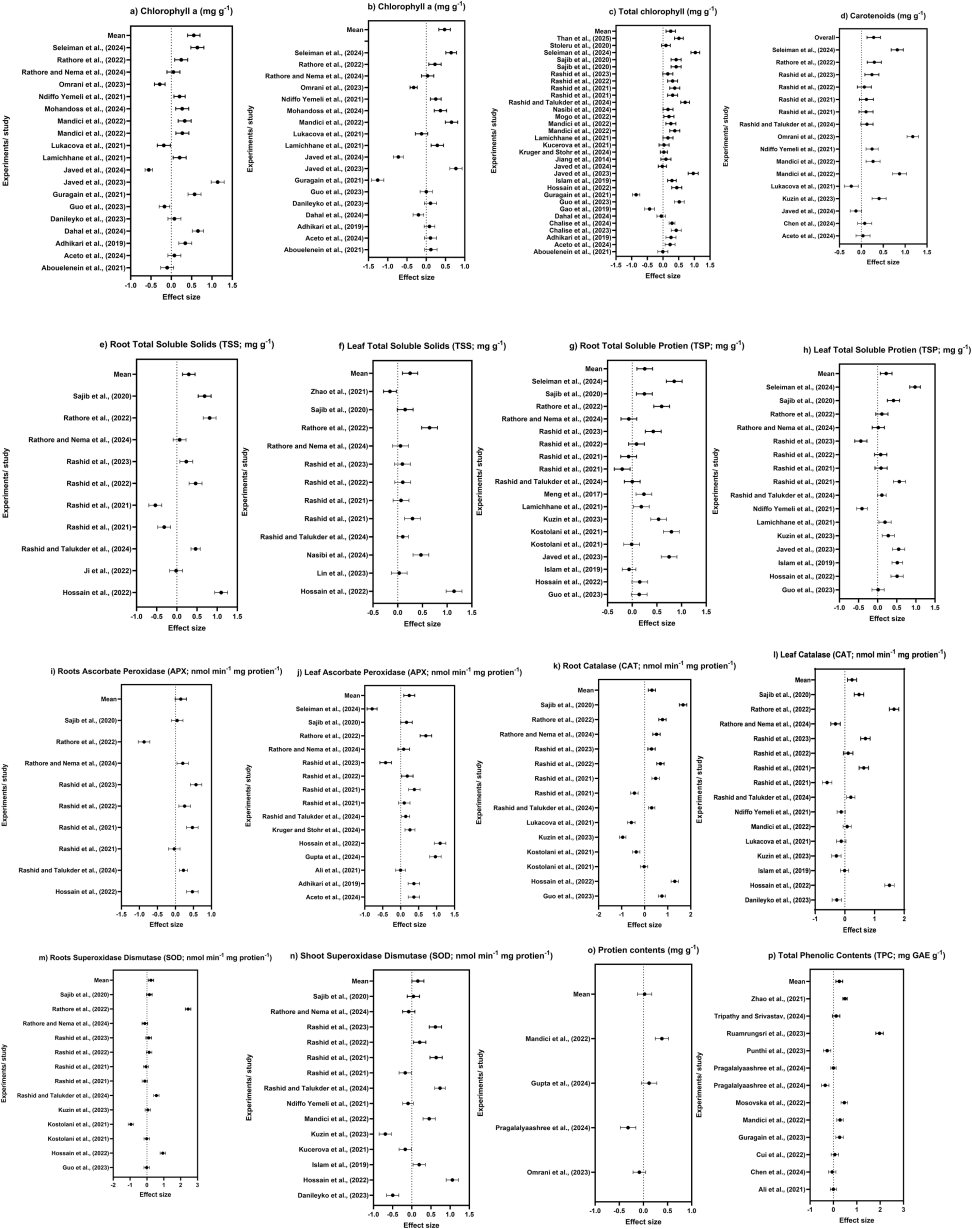
**(a)** Chlorophyll a, **(b)** chlorophyll b, **(c)** total chlorophyll, **(d)** carotenoids, **(e)** root total soluble solids, **(f)** leaf total soluble solids, **(g)** root total soluble protein, **(h)** leaf total soluble protein, **(i)** root ascorbate peroxidase, **(j)** leaf ascorbate peroxidase, **(k)** root catalase, **(l)** leaf catalase, **(m)** root superoxidase dismutase, **(n)** shoot superoxidase dismutase, **(o)** protein contents, and **(p)** total phenolic content effect sizes expressed as log response ratios (LnRR) with 95% confidence interval comparing PAW application effects relative to control treatment.

**Figure 5:**
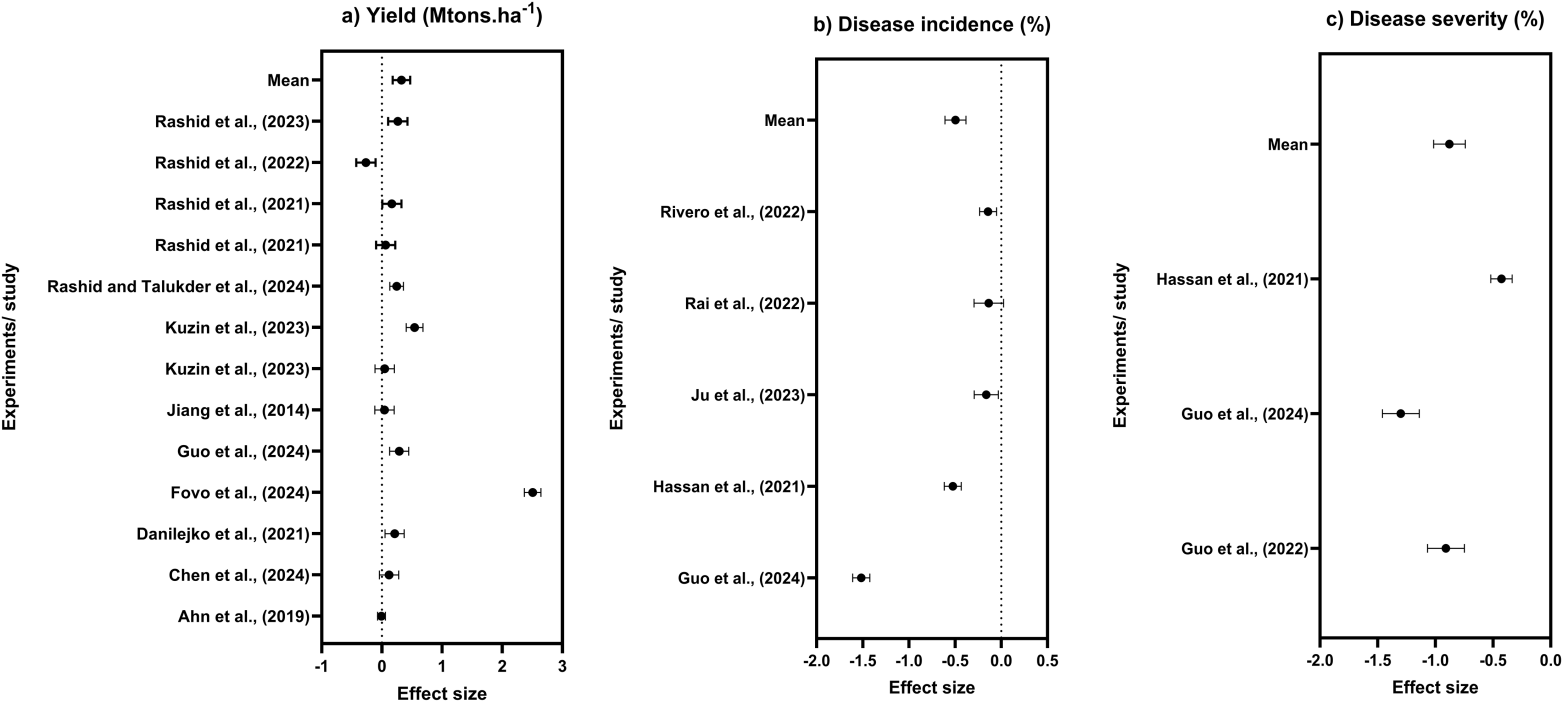
**(a)** Yield, **(b)** disease incidence, and **(c)** disease severity effect sizes expressed as log response ratios (LnRR) with 95% confidence interval comparing PAW application effects relative to control treatment.

Papers were included in meta-analysis based on an inclusion criterion such as having control treatment with normal water, sample size > 2 and containing the plant or seedling germination and growth parameters, yield components, yield, chlorophyll attributes, quality traits, water quality, and disease incidence. Review papers, duplicated research and overlapping results, insufficient data, conference abstracts, and focus on food and medicines were excluded. Data from the screened papers were extracted from the results presented in tables and figures. Among the papers searched for meta-analysis (2,838), 164 papers were selected based on available data for meta-analysis. However, the number of papers contributing parameter-specific data varies for **Figures 2**-**5** due to available statistical information for respective parameters. Overview of the studies included in meta-analysis are presented in **Supplementary Table 4**. The geographical locations of the experimental sites are represented in **Figure 2**. For each location and spatial distribution statistics of the papers published, country names along with publication number show that approximately 25 papers were published from China followed by Korea, Thailand, Iran, and USA, and Uzbekistan had the minimum number of publications (**Supplementary Figure 1a**). The temporal distribution of papers published indicated that PAW application in agriculture is increasing with time as clear from the annual published papers trend (**Supplementary Figure 1b**). The keywords co-occurrence network generated using VOSviewer illustrates the linkages and clustering within the PAW irrigation research domain (**Supplementary Figure 2a**).

#### 2.1.2 Screening process

Different research papers were searched for ensuring robust and pertinent literature based on the objectives. Many articles were obtained by initial search and were screened for duplication and irrelevancy by checking the relevance through the title and abstract and thereafter followed by a full text search to ensure the reliability and relevance of the articles for inclusion in meta-analysis.

### 2.2 Treatment selection and grouping

Data were extracted from all the selected papers, and for meta-analysis, two treatments, i.e., untreated control and the optimum recommended PAW treatment among all treatments in each study, were selected. To ensure comparability across diverse designs, treatment grouping was carried out to standardize PAW categories to avoid the inconsistent naming and terminology used. The dataset consisted of multiple response variables covering early establishment, growth, yield, biochemical characteristics, disease-related parameters, and water properties. The included papers contain treatments including PAW applied through irrigation, pot experiments, seed priming, and foliar application. To ensure the comparison across multiple studies, the variables reported in different units and scales were harmonized prior to effect size calculations. Measurements reported in different units and scales were converted to common units using standard conversion factors. The indices as % were used for analysis to avoid scaled induce bias and to ensure that effect size calculations are based on standardized and comparable metrics across all the included parameters and studies in meta-analysis. Restricting the analysis to control and optimum PAW treatments across all the included studies provided a uniform basis for comparison with respective effect sizes and statistical evaluations. Because of non-availability of sufficient data, moderator analysis was not performed to avoid unstable estimates and overfitting, pooled effect was interpreted with quantitative discussion on heterogeneity.

### 2.3 Analytical framework for meta-analysis

For all extracted data related to plant establishment, growth parameters, pigment concentration, antioxidant activity, yield, and water quality, effect sizes (Equation 1) were computed using the natural logarithm of the response ratio (LnRR). This metric was derived by comparing the mean values of each variable in the PAW-treated group to those in the control group, providing a standardized measure of treatment impact.

(1) $$lnR\;=ln\;(\frac{xi}{xc})=lnxi\;-\ln xc$$

In each study, the effect size was calculated using natural logarithm of the response ratio (LnRR), where *xi* represents treatment means and *xc* denotes control treatment means for the respective study. For studies where standard deviation (SD) was not given, SD = SE √(*n*) from standard error (SE) and number of replicates (*n*). The variance of log response ratio (VLnR) was calculated using the formula as follows (see Equation 2) to account for sampling variability following the approach described by Benítez-López et al. (2017).

(2) $$VLnR=\frac{Si^{2}}{ni.\;xi^{2}}+\frac{Sc^{2}}{nc.\;xc^{2}}$$

where *Si* and *Sc* are treatment and control groups’ SD, respectively, while *ni* and *nc* show its corresponding sample sizes. In addition to individual estimates, the overall effect size was estimated as pooled log response ratio (LnRR ++) with 95% confidence intervals (CIs). Each study weight (*wi*) (Equation 3), overall effect size (LnRR ++) (Equation 4), and 95% CI (Equation 5) were obtained as follows:

(3) $$Wi\;=\frac{1}{Vi}$$

(4) $${LnRR}_{++}=\frac{\sum wi\text{Ln}Ri}{\sum wi\;}$$

(5) $$95\%\;\text{CI}\;={LnRR}_{++}\pm 1.96\;x\;\sqrt{\frac{1}{\sum wi}}$$

where *Vi* represents the combined variance, including both the within-study variance and the between-study variance. Furthermore, the mean effect size (MES) of all the included parameters was calculated as percentage change using Equation 6.

(6) $$\%\;Change=(e^{LnRR}-1)\;x\;100$$

Certain parameters like nitrate, nitrite, and H_2_O_2_ were analyzed through mean differences as LnRR determination affected the results. Because pH is a logarithmic variable (−log_10_[H^+^]) and LnRR calculation was inappropriate, pH across the studies was analyzed as the mean difference in pH units.

Data were extracted from the selected papers included in meta-analysis from tables, and if the data were presented in figures, then data were obtained through Get Data Graph Digitizer software version 2.26 (https://getdata-graph-digitizer.software.informer.com). Data curation, merging, grouping, optimum PAW treatment selection, and statistical analysis were performed in Microsoft Excel, Metafor package for data analysis (Viechtbauer, 2010). The figures were made through GraphPad Prism 10 software version 10.6.0 (Motulsky, 2007). To assess the overall effect, pooled effect calculated at 95% CI and the estimates with their respective CI were plotted using forest plots. Deviation of the effect size from the null line for each parameter showed the corresponding negative and positive impact under PAW application.

## 3 Results

### 3.1 Overview of dataset

The dataset compiled for this meta-analysis was extracted from peer-reviewed studies investigating the effect of PAW on plant establishment, growth, biochemical traits, and disease resistance. The dataset contained different effect sizes extracted across key response categories, including germination percentage, growth traits (plant height, stem diameter, root and shoot biomass, leaf number, and leaf area), photosynthetic pigments (chlorophyll *a*, chlorophyll *b*, total chlorophyll, and carotenoids), biochemical traits (soluble sugars, proteins, and antioxidant activity), and disease-related parameters (incidence and severity). In addition, physicochemical properties of PAW (pH, EC, nitrate, hydrogen peroxide, and ORP) were recorded when reported. Effect sizes were calculated as log response ratios (LnRR) of treatment relative to control, and studies were weighted by their variance to account for differences in sample size and experimental precision. Collectively, this dataset provides a comprehensive quantitative basis to evaluate the agronomic and biochemical impacts of PAW across multiple crops and production systems.

### 3.2 Plasma generation methods and reactor types

Although plasma generation methods and reactor types were not directly included in the meta-analysis, their distribution was documented based on the mechanism to provide an overview of the diversity of systems employed in the literature. The reported configurations included the most commonly used one as DBD plasma systems (DBDPS) followed by pulsed jet systems (PJS), gliding arc discharge plasma systems (GADPS), glow discharge plasma systems (GDPS), tubular and surface discharge plasma systems (TS & SDPS), atmospheric discharge plasma systems (ADPS), arc and hybrid plasma systems (AP & HPS), electrolytic plasma systems (EPS), and pin- and rotating-bed plasma systems (P & RBPS). These categories are plotted in **Supplementary Figure 2b** to illustrate the frequency of use across studies, reflecting the wide range of plasma sources applied for PAW generation in agricultural research. While not directly analyzed, this descriptive classification underscores the technological variability that underpins PAW studies and highlights the need for greater standardization in future applications.

### 3.3 Parameterization of discharge systems for plasma-activated water synthesis

PAW has been generated using diverse discharge configurations with wide-ranging operational parameters (**Supplementary Table 1**). Arc discharges typically operate at 3–30 kV and 50 Hz–40 kHz, for 30 s to 10 min. Hybrid plasma integrates DBD plasma with gas–liquid interaction to enhance RONS. Atmospheric and hybrid plasmas span 150 V to 347 kV, 0.05–18 kHz, with treatment times up to 1 h. Dielectric barrier discharge (DBD) systems exhibit the broadest range, from 40 V to 160 kV, 0.3 Hz to 120 MHz, and 15 s to 1 h exposure. Electrochemical discharges operate at 300 V to 15.8 kV, ≤ 440 kHz, often for several hours. Gliding arc discharges range from 1.5 to 24 kV, 30 Hz to 900 kHz, for 30 s to 90 min, whereas glow discharges use 250 V to 10 kV, 0.11–440 kHz. Plasma jets function between 35 V and 20 kV across Hz–kHz–MHz domains, with 1–60 min treatments. Pulsed and spark discharges operate at 4.6–20 kV, 1 Hz to 15 kHz, for durations from seconds to 1 h.

### 3.4 PAW for plant establishment and growth

Application of PAW demonstrated a consistent positive influence on plant establishment, vegetative growth, and phenological development. The germination percentage, germination rate, root length, and shoot length effect sizes expressed as log response ratios (LnRR) with 95% CI comparing PAW application effects relative to control treatment are shown in **Figure 3**. Germination (%) meta-analysis (**Figure 3a**) demonstrated that PAW application exhibited overall positive effect relative to control. Across the individual studies, most of the log response ratios (LnRR) estimates were observed greater than zero, indicating an increase in germination percentage under PAW application. Substantial heterogeneity was observed among the included studies; however, pooled effect size showed a moderate to large overall effect. The overall effect size was positive with LnRR = 0.087 (95% CI: −0.05 to 0.22 and *p* = 1) showing 13% improvement in germination percentage and considerable heterogeneity was detected (*I*^2^ = 90.1%) supporting the influence of PAW across different studies. **Figure 3b** represents the germination rate of different plants subjected to PAW, which revealed that PAW application improved the overall germination rate across all the plant species. The pooled effect size was statistically significant (LnRR = 0.08, 95% CI: 0.006 to 0.16, *p* = 0.004), corresponding to an estimated 19% increase in germination rate compared to control, with heterogeneity (*I*^2^ = 90%) indicating variability across studies.

The root length is also affected by the application of PAW as presented in **Figure 3c**. A heterogeneous response of root length to PAW application revealed positive log response ratio values, which indicate increased root length compared to control treatment. The meta-analysis indicated a significant positive effect, with the pooled LnRR of 0.18 translating to an approximate 27% increase in root length relative to control (95% CI: 0.03 to 0.33, *p* = 0.03; *I*^2^ = 96%), indicating variation in studies. The magnitude of variation differed across the study due to crop species, PAW generation parameters, and soil and environmental conditions. The overall pattern suggests a consistent trend in root growth under PAW treatments. The forest plot (**Figure 3d**) illustrates the variation in LnRR for shoots length across individual studies. Almost all the studies are positioned at the right side of the no line effect showing PAW potential in enhancing plant growth. The narrow CI that did not overlap with zero indicates statistical robustness while others with a wider CI exhibited less precise estimate while others showed minimum response with no consistent negative response. A significant pooled LnRR of 0.25 was observed (95% CI: 0.09 to 0.39, *p* = 0.001), corresponding to about a 39% improvement compared with control, despite observed heterogeneity (*I*^2^ = 96%).

Analysis indicated that PAW had a positive effect on the fresh and dry weight/biomass accumulation of roots. Roots’ fresh weight forest plot (**Figure 3e**) revealed that most of the studies’ LnRR values lie above zero, showing an increase in biomass accumulation in the plant root system compared to the control. The pooled analysis showed a significant positive effect (LnRR = 0.29), equivalent to a 59% increase in fresh roots biomass compared to control (95% CI: 0.14 to 0.44, *p* < 0.001), with heterogeneity measured at *I*^2^ = 98%. Similarly, roots’ dry weight (**Figure 3f**) exhibited a significant effect (*p* < 0.05) with studies showing enhanced root biomass under PAW application. The majority of root dry weight LnRR lie on the positive side. Overall, the pooled LnRR was 0.27, indicating a statistical significance with 52% increase relative to control (95% CI: 0.12 to 0.42, *p* < 0.004); however, heterogeneity remained evident (*I*^2^ = 97%). The overall pooled LnRR of root fresh and dry weight confirmed that PAW application significantly improved root growth and shoot growth, which signifies the potential of PAW application in enhancing belowground biomass and improves plant establishment.

PAW application had a significant influence on plants’ aboveground biomass. Seedling fresh weight exhibited a significant positive effect under PAW application as shown in **Figure 3g**. A significant positive effect was detected for seedling fresh weight, with LnRR = 0.26 (34% increase; 95% CI: 0.12–0.41, *p* < 0.001; *I*^2^ = 94%). All the experimental studies showed narrow CIs reflecting the favorable overall effect. The pooled effects showed 34% increase in seedling fresh weight under PAW application compared to control. The seedling dry weight also exhibited a significant (*p* < 0.005) positive effect with studies showing enhanced aboveground biomass under PAW application as shown in **Figure 3h**. All the studies demonstrated dry biomass accumulation in seedlings indicating stronger structural development and carbon allocation in plants. For all the studies, seedling dry weight LnRR were observed on the positive side of the forest plot reflecting a significant positive influence on plant biomass accumulation under PAW application over the control treatment. The overall pooled effect size of seedling dry weight confirmed the PAW application’s significant influence on plant growth and early establishment. The pooled effect for seedling dry weight was significant (LnRR = 0.19, 95% CI: 0.03–0.35, *p* = 0.017), showing a 28% increase over control with moderate heterogeneity (*I*^2^ = 90%).

A consistent positive response was revealed for vegetative and growth parameters of the plants under PAW application (**Figures 3i–l**). **Figure 3i** suggests that PAW application exerted a positive influence on plant height. Most of the studies’ LnRR were found at the positive side, which reflects enhanced vigor and growth under PAW application over the control. The analysis revealed that a significant pooled effect size for plant height was positive (LnRR = 0.076, 95% CI: −0.053 to 0.205, *p* = 0.249); however, the CI overlapped zero, and its effect was statistically non-significant as shown in **Figure 3i**. A moderate to substantial heterogeneity was observed in different studies (*I*^2^ = 74%). Almost all the studies showed a positive effect size compared to control. Meta-analysis showed a significant positive outcome (LnRR = 0.28), about a 42% increase in stem diameter (**Figure 3j**) relative under PAW application to control (95% CI: 0.14–0.42, *p* < 0.001; *I*^2^ = 97%). Leaf number and leaf area under PAW application are presented in **Figures 3k, l**, respectively. Both the leaf number and leaf area showed a significant positive (*p* < 0.05) effect when subjected to PAW application compared to control treatment. The leaf number per plant was observed higher under PAW application compared to untreated control, which indicates accelerated leaf initiation and canopy development. Similarly, the leaf area positive response resulted in photosynthetic surface area expansion under PAW treatments, which increases light interception and biomass accumulation in plants. The pooled effect for leaf number was significant (LnRR = 0.48, 95% CI: 0.346–0.618, *p* < 0.001), corresponding to an estimated 76% increase over the control, while root length also showed a significant improvement of 68% (LnRR = 0.416, 95% CI: 0.273–0.558, *p* < 0.001) under PAW application, though heterogeneity was high for both (*I*^2^ > 80%).

### 3.5 PAW role in plant chemistry/pigments, biochemicals, and antioxidants

PAW application significantly influences plant biochemical composition by modulating pigments, primary metabolites, and antioxidant defense systems. PAW application enhances chlorophyll content and carotenoids, which improve light use efficiency and energy capture. Furthermore, PAW application stimulates soluble protein accumulation and phenolic compounds, which help in secondary metabolism and stress adaptation. The RONS in PAW activates enzymatic antioxidants including superoxide dismutase (SOD), catalase (CAT), and ascorbate peroxidase (APX), which reduces the oxidative damage and maintains cellular homeostasis. The forest plot shown in **Figure 4** demonstrated that PAW application had a significant positive influence on plants’ pigments accumulation. The chlorophyl contents were significantly affected by PAW application (**Figure 4a**). Majority of the studies lying on the positive side suggest that PAW application enhanced chlorophyll a content in plants, favoring photosynthetic efficiency. A significant positive effect was detected for chlorophyll a with LnRR = 0.55 (32% increase; 95% CI: 0.401–0.704, *p* < 0.001; *I*^2^ = 85%). Chlorophyll b content (**Figure 4b**) followed a trend similar to chlorophyll for the included studies. Most of the studies’ positive LnRR suggested that PAW application enhances photosynthetic efficiency. The pooled LnRR was 0.475, which showed that PAW application significantly (17%) increased chlorophyll b compared to control (95% CI: 0.326–0.625, *p* < 0.001), though heterogeneity was present (*I*^2^ = 99%). Total chlorophyll content showed consistent positive response for all the studies as shown in **Figure 4c**. The pooled effect was significant (LnRR = 0.24, 95% CI: 0.096–0.396, *p* = 0.001), showing a 35% increase over control with moderate heterogeneity (*I*^2^ = 97%). Leaves’ carotenoid contents (**Figure 4d**) are increased under PAW application, with all the studies exhibiting positive LnRR, which highlights PAW’s role in promoting photosynthetic pigments profile in plant species, thereby supporting light harvesting and stress resilience. Meta-analysis showed PAW application’s significant positive outcome (LnRR = 0.281), about a 38% increase relative to control (95% CI: 0.13–0.43, *p* < 0.001; *I*^2^ = 97%).

Root total soluble solids (TSS) are significantly influenced by PAW application as shown in **Figure 4e**. Most of the studies that exhibited positive LnRR suggested that PAW application enhances the TSS in roots. An increase of 50% for TSS in roots under PAW application reflects that PAW application enhances roots’ biochemical accumulations. **Figure 4f** reveals that TSS in plant leaves exhibited a similar trend and maintained a positive LnRR. The overall effect size suggested that leaf TSS was increased by 41% under PAW application over the control. Likewise, roots and shoots’ total soluble protein (TSP) depicted a clear positive increase under PAW application for all the studies as shown in **Figures 4g, h**. The pooled analysis showed that PAW application increased root TSS (60%), shoot TSS (40%), root TSP (36%), and shoot TSP (33%) compared to control with high heterogeneity across all parameters (*I*^2^ > 80%) contributing to growth, metabolic regulation, and stress adaptation.

**Figure 4** demonstrated that PAW application had a significant positive influence on APX and catalase (CAT) accumulation in roots and shoots. These biochemicals were significantly affected by PAW application as majority of the studies exhibits positive LnRR. Root APX were significantly influenced by PAW application as shown in **Figure 4i**.

PAW application was non-significant for root APX with LnRR = 0.15 (95% CI: −0.006 to 0.31, *p* = 0.059; *I*^2^ = 98%). Leaf APX content exhibited a similar trend and maintained a positive LnRR (**Figure 4j**). The pooled LnRR was 0.34, which showed that PAW application significantly (42%) increased leaf APX content (95% CI: 0.18–0.49, *p* < 0.001) compared to control (**Figure 4j**), though heterogeneity was present (*I*^2^ = 97%). Likewise, root and shoot CAT content (**Figures 4k, l**) depicted a clear positive increase under PAW application for all the studies. The pooled effect for root CAT content was significant (LnRR = 0.24, 95% CI: 0.088–0.397, *p* < 0.001), corresponding to an estimated 29% increase over the control, while leaf CAT content also showed a significant improvement of 35% (LnRR = 0.314, 95% CI: 0.16–0.47, *p* < 0.001) under PAW application, contributing to growth, carbon storage, and metabolic capacity, though heterogeneity was high for both (*I*^2^ > 80%).

**Figure 4** reveals that PAW application had a significant influence on SOD content, protein content, and TPC accumulation in plants. A significant influence of PAW application was noted with positive effect sizes, confirming PAW’s potential. Root and shoot SOD content were significantly influenced by PAW application as shown in **Figure 4m, n**. The pooled effect for root SOD content was significant (LnRR = 0.23, 95% CI: 0.07–0.39, *p* = 0.003), corresponding to an estimated 15% increase, while shoot SOD content revealed a positive effect (LnRR = 0.15, 95% CI: 0.002–0.31, *p* = 0.047) under PAW application. Likewise, protein content and TPC (**Figures 4o, p**) depicted a positive increase in LnRR under PAW application for all the studies. The overall means showed that PAW application improved protein content by 6% and TPC by 13%.

### 3.6 PAW effects on crop health and disease resistance

Studies with available data for yield of different crops under PAW application were analyzed and shown in a forest plot (**Figure 5a**). Most studies are clustered at the positive side of the axis, confirming the beneficial effects of PAW on crop yield. Few studies resulted in modest or negligible response, but no consistent negative effect was noted. A pooled LnRR of 0.32 was observed, indicating a potential 17% increase in yield (95% CI: 0.18 to 0.47, *p* < 0.001) and heterogeneity was high (*I*^2^ > 90%). Analysis demonstrated that PAW applications improved crop health by lowering disease incidence and enhancing plant resistance against the pathogens. PAW reduces the disease incidence in plants due to its antimicrobial potential in agricultural systems. PAW enriched with RONS has been shown to suppress pathogen proliferation and dissemination, thereby lowering the risk of disease infection. Across individual studies, the LnRR for disease incidence was positioned on the negative side of the axis, confirming a lower level of infection in PAW application compared to control treatments (**Figure 5b**). The overall LnRR revealed that PAW application decreased disease incidence by 31% (LnRR = −0.47; 95% CI: −0.61 to −0.38, *p* < 0.001) in plants over the control treatment. A clear potential of PAW application in the mitigation of plant disease severity is shown in **Figure 5c**. The forest plot indicated that all the studies showed effect size with negative values ranging from −0.04 to −1.4 with an LnRR of −0.878 (*p* < 0.001). All the LnRR estimates fall below zero, indicating a consistent reduction in disease severity in plants. A summary of studies evaluating PAW against plant pathogens is presented in **Supplementary Table 2**. Across a range of crops, PAW demonstrated inhibitory effects on diverse fungal and bacterial diseases, including seed-borne and foliar pathogens. The compiled evidence indicates that PAW applications consistently reduced pathogen load or disease severity, though the extent of effectiveness varied with crop species, pathogen type, and treatment protocol.

### 3.7 Plasma-driven water properties

**Figure 6:**
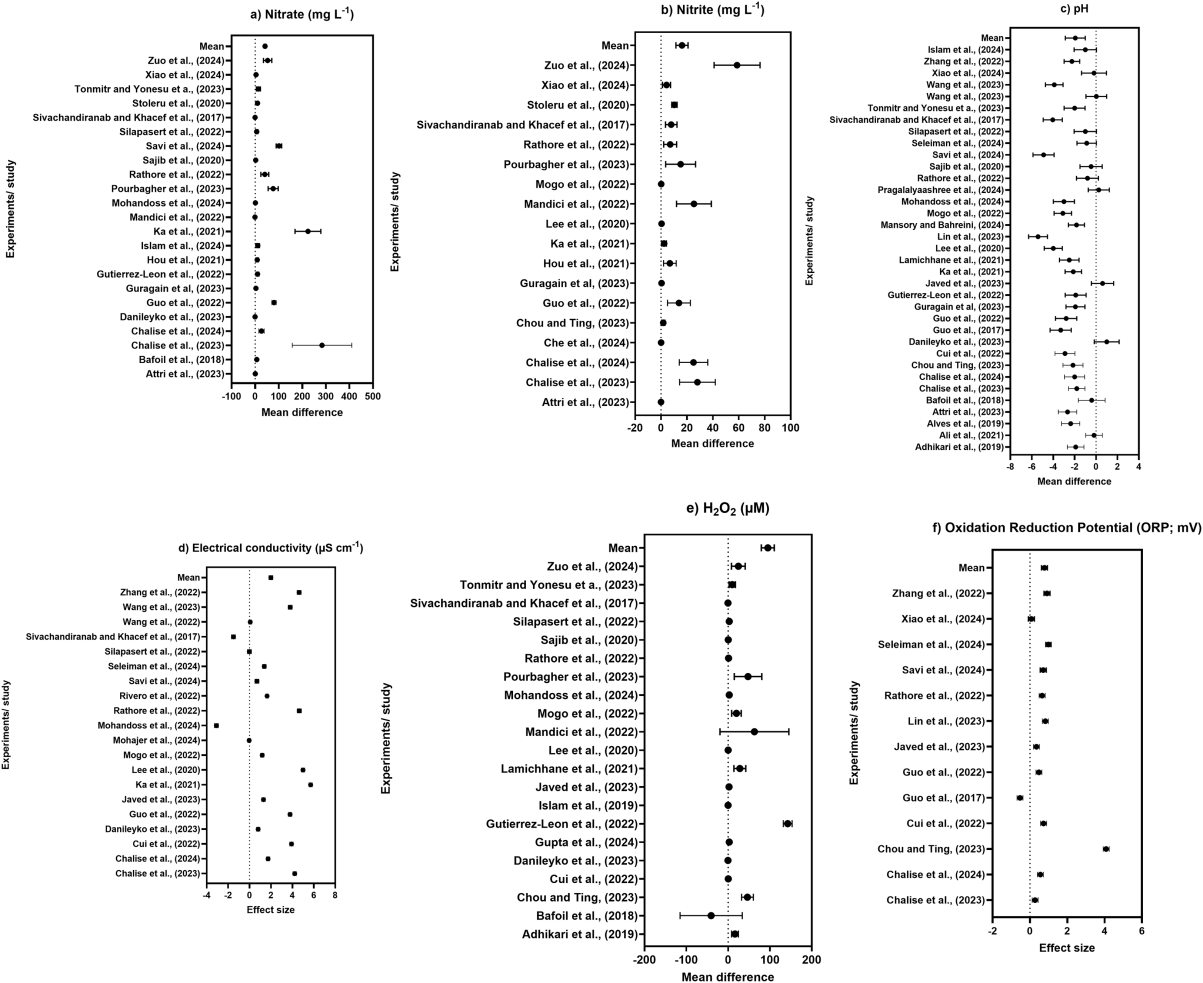
**(a)** Nitrate (mg L^−1^), **(b)** nitrite (mg L^−1^), **(c)** pH, **(d)** electrical conductivity (µS cm^−1^), **(e)** H_2_O_2_ (µM), and **(f)** oxidation–reduction potential (mV) effect sizes expressed as log response ratios (LnRR) with 95% confidence interval comparing PAW application effects relative to control treatment.

The meta-analysis in **Figure 6a** summarizes the plasma activation effect on nitrate contents of water across different studies. All the studies’ mean differences fall on the positive side of the null line effect, confirming the plasma activation improvement on the nitrate content of water. Plasma activation had a strong positive effect on the nitrate content of water across the evaluated studies. All studies lie on the positive side of the null line, and the pooled analysis confirmed that plasma activation significantly enhanced nitrate accumulation relative to control (pooled mean difference = 12.23, 95% CI: 11.21–13.26), indicating an increase under PAW treatment though heterogeneity was observed across the studies. **Figure 6b** shows the nitrite content in water influenced by plasma activation across different studies. All the studies are clustered on the positive side of the axis, stating the potential of higher nitrite content in water with plasma activation. The heterogeneity was found higher among the studies and a random effect model resulted in a pooled difference of 4.05 (95% CI: 3.75–4.36), indicating a statistically positive effect. A substantial decrease in pH with plasma activation is shown in **Figure 6c** with pooled mean difference (−2.02) compared to control, indicating strong acidification of water induced by plasma treatment. Direction of the effect on pH was consistently negative with more heterogeneity (*I*^2^ = 84%) depending on plasma configurations and activation parameters. Across all the studies, the EC of water was significantly (*p* < 0.05) affected by plasma activation, as shown in **Figure 6d**. All the log response ratios fall on the positive side of the axis. The pooled estimates (LnRR = 1.99, 95% CI: 1.84–2.14, *p* < 0.001, *I*^2^ >90%) showed a significant rise in EC of water, which reflects that reactive ionic species are generated during plasma activation. Plasma activation markedly increased the hydrogen peroxide concentrations across all the studies as shown in **Figure 6e**. All the effect sizes were consistently positive, confirming that plasma activation enriched the solution with hydrogen peroxide compared to the control. High heterogeneity shows substantial variability across the studies, and the pooled mean difference was 12.37 with 95% CI (10.77–13.78), demonstrating a statistically positive effect despite between-study variations confirming an increase in hydrogen peroxide ions in water through plasma activation. **Figure 6f** shows the ORP of water induced by plasma activation across different studies. All the studies showed a significant positive and consistent ORP, confirming that plasma activation shifted the redox balance toward a more oxidizing environment relative to control. Although the magnitude of each study differed, the pooled effect size (LnRR = 0.77, 95% CI: 0.62–0.93, *p* < 0.001, *I*^2^ >90%) confirmed a significant increase in ORP.

## 4 Discussion

This meta-analysis provides quantitative evidence about the PAW’s significant potential as a sustainable irrigation input in modern agriculture. Analyzing multiple crops’ parameters and experimental conditions, consistent positive impacts on plant establishment, growth, and yield were observed. These outcomes are aligned with the PAW unique chemistry and the presence of reactive species, which act as biostimulants and as protective agents. A consistent trend across studies demonstrates that PAW significantly enhances seed germination rate, germination percentage, root/shoot fresh and dry weight, leaf area and stem diameter, and early plant growth. The germination rate of barley is improved with PAW application, as reported by Seleiman et al. (2024). This improvement in plant growth parameters is attributed to nitrate enrichment, which serves as a nutrient and growth signal, while reactive oxygen species (H_2_O_2_) act as low-level stimulants for cell division and elongation. The positive effects of PAW application are seen in various crops showing increased shoot/root dry length, chlorophyll content, vigor indices, and fresh/dry biomass (Guo et al., 2021). Yield and productivity of the plants are also enhanced by PAW application (Seleiman et al., 2024). Improved seedling establishments often translate into higher crop yields and productivity (Antoni et al., 2025). Several studies confirm increased yields in rice (17%), tomato (three times), radish (50%), wheat, and other crops when irrigated or primed with PAW (Jirešová et al., 2022; Priatama et al., 2025; Rashid et al., 2022). These gains are attributed to physiological stimulation such as enhanced nutrient uptake, improved chlorophyll synthesis, and greater biomass accumulation in tissues (Guo et al., 2021; Karimi et al., 2024).

Biochemical traits of the plants, including soluble sugars, proteins, phenols, ascorbates, and photosynthetic pigments such as chlorophyll a, chlorophyll b, total chlorophyll, and carotenoid contents, are significantly improved with PAW application. PAW-treated plants elevated the biochemical traits and photosynthetic pigments (Aceto et al., 2024; Veerana et al., 2024). PAW positively influences plant nutritional parameters by improving chlorophyll content and facilitating nitrogen uptake. Improved chlorophyll, soluble protein content, and antioxidant enzyme activity following PAW treatment are reported by Aceto et al. (2024). PAW positively influences plant nutritional parameters by improving chlorophyll content and facilitating nitrogen uptake. PAW and foliar nutrition boost photosynthetic activity and productivity (Rathore and Nema, 2025). Plasma-generated nitrate in PAW has been suggested as a safer nitrogen source compared to conventional fertilizers (Andrade et al., 2025). This PAW is rich in RONS, which can enhance plant pigments including chlorophyll a/b and carotenoids and biochemical traits such as antioxidant enzyme activity, soluble proteins, and sugars by triggering mild oxidative signaling (Rashid et al., 2021; Chalise et al., 2024). These benefits show PAW’s capability of photosynthesis, nutrient assimilation, and stress tolerance improvements in plants (Rashid et al., 2021; Kuzin et al., 2023), which vary by crop, PAW chemistry, and dose while excessive RONS may cause pigment loss or oxidative damage. Therefore, carefully parameterized PAW in terms of RONS, pH, and application rates demonstrates its potential to enhance crop productivity and quality under soil- and crop-specific conditions.

Beyond growth promotion, PAW has been effective in managing pathogens. It reduced severity of late blight in potatoes and scab in apples to levels comparable with fungicides (Kuzin et al., 2024; Gao et al., 2025). Postharvest storage tests of PAW showed inhibition of bacteria and fungi, and the microbial safety of produce like peanut sprouts and leafy greens was enhanced (Malahlela et al., 2024). PAW contributes to stress mitigation by regulating antioxidant enzyme systems, stimulating cellular changes such as parenchyma expansion, and modulating immune responses (Aceto et al., 2024). It enhances root and shoot development under abiotic stresses, including low temperature and nutrient deficiency (Veerana et al., 2024). It also improves reproductive traits such as tuber number and weight (Rashid et al., 2022). PAW, enriched with RONS, improves plant health by directly inactivating pathogens on surfaces and by inducing systemic resistance. Its oxidative chemistry (e.g., H_2_O_2_, NO_2_^-^, and OH) damages microbial membranes and DNA, while simultaneously triggering defense pathways such as salicylic acid, jasmonic acid, and ethylene signaling. Applications in tomato have reduced *Xanthomonas vesicatoria* leaf spot severity by up to 61% without direct in-plant bactericidal effects, indicating priming of host immunity (Pérez-Pizá et al., 2019). PAW suppressed disease symptoms and limited pathogen proliferation (Zambon et al., 2020). Broader studies show enhanced phenylalanine ammonia-lyase activity, increased antioxidants, and overall improved vigor under both biotic and abiotic stress (Adhikari et al., 2020).

PAW is generated by exposing water to non-thermal atmospheric plasma via plasma jets, DBD, or corona discharge resulting in the dissolution of RONS such as H_2_O_2_, O_3_, OH, O_2_^-^, NO_2_^-^, NO_3_^-^, and ONOOH into the liquid phase (Wong et al., 2023; Scholtz et al., 2025). These species, formed through plasma–gas–liquid interactions, are accompanied by acidification (pH ~ 2–4) due to nitric and nitrous acid formation, increased EC from dissolved ions, and elevated ORP immediately after activation. Short-lived radicals (OH, O_2_^-^) drive immediate reactivity, while long-lived species (H_2_O_2_, NO_3_^-^) confer residual bioactivity, enabling antimicrobial and plant-modulating effects even after storage. Plasma activation also alters physical properties, which lower surface tension, reduce contact angles, and slightly increase viscosity at higher temperatures, which improves wetting, spreadability, and penetration on plant surfaces (Shaji et al., 2023). The exact chemical profile and stability of PAW depend on plasma power, feed gas composition, activation time, and storage conditions, with decay kinetics of RONS influencing its shelf life and efficacy. These combined chemical and physical modifications underpin PAW’s multifunctional role in agriculture, food safety, and environmental applications, while highlighting the need for standardized generation protocols to ensure reproducible properties across studies (Ma et al., 2015; Sivachandiran and Khacef, 2017; Thirumdas et al., 2018).

## 5 Scaling-up PAW for irrigation: properties, limitations, and future directions

**Figure 7:**
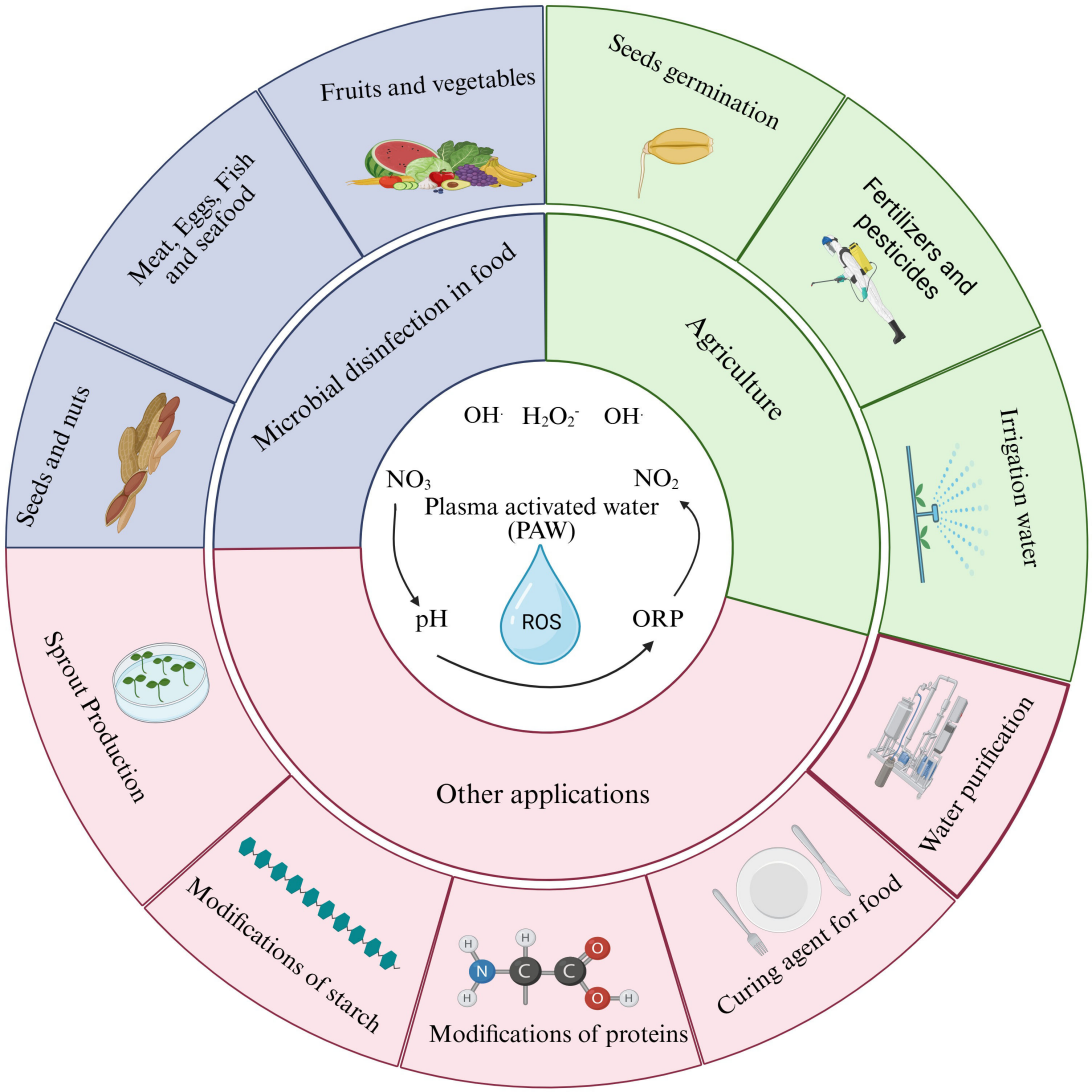
Applications of PAW across food, agriculture, and environmental sectors. PAW exhibits antimicrobial properties, enhances seed germination and fertilizer efficacy, and facilitates protein/starch modification, pesticide degradation, and water purification through reactive species-driven processes.

The technological and engineering considerations are discussed in this section for scaling of PAW systems. The following perspective extends beyond the meta-analysis outcomes and are stated to outline implementation pathways rather than data-supported conclusion. PAW is used in agriculture across multiple domains. PAW has been used for microbial disinfection in food (meat, eggs, seafoods, fruits, vegetables, seeds, and nuts) and other processes. In the agriculture sector, it is used for seed germination enhancement, seedling growth, fertilizer management, and pesticide degradation. Other applications of PAW include sprouts production, modification of starch and proteins, and a curing agent for food and water purification (**Figure 7**). Plasma is made through direct and indirect treatments.

Direct plasma treatment involves the simultaneous bombardment of target surfaces by charged particles, RONS, and UV photons (**Supplementary Figure 3a**). This synergistic interaction facilitates efficient sterilization, surface functionalization, pathogen inactivation, and rapid physicochemical modification. However, the high energy density associated with direct exposure can induce structural damage and restrict treatment efficacy to regions directly exposed to plasma. In contrast, indirect plasma treatment relies on long-lived RONS without direct contact with high-energy plasma ions or electrons. This modality offers a gentler and more spatially uniform treatment, making it particularly suitable for large-scale or thermally sensitive substrates. In PAW systems, the photons’ effects are transient and only act at the activation stage while the biological effects are mediated by dissolved RONS. Key applications include seed priming, the synthesis of PAW, and the treatment of geometrically complex or fragile surfaces. Although the reactivity of indirect plasma is comparatively lower and the process slower, its enhanced scalability and reduced risk of sample degradation render it highly advantageous for agricultural systems, especially in irrigation and crop production frameworks. Literature indicates that PAW can be generated at low cost with high efficiency across different reactor systems and scales. Scholtz et al. (2025) estimated USD 0.012 L^−1^ using microbubble discharge to USD 0.315 L^−1^ with transient spark corona discharge. The PAW system process costs USD 0.50 per ton of produce (Misra et al., 2024) and approximately 0.02–0.03 kWh L^-^¹ energy use is reported by Kooshki et al. (2024). Tuning RONS in PAW leads to 30% reduction in total costs (Kooshki et al., 2024) and nano pulsed plasma microbubbles exhibited a high energy yield (82.1 g/kWh) and a low energy cost (0.25 kWh/m^3^) (Meropoulis and Aggelopoulos, 2023).

PAW is strongly influenced by source water quality with both salinity and iron contents. Higher EC in saline or brackish water alters plasma discharge and promotes reactive chlorine species formation (HOCl, chlorite, chlorate, and perchlorate); bromide can yield bromate (Lukes et al., 2014; Silverj et al., 2025). Excessive iron in groundwater source rapidly consumes H_2_O_2_ in PAW via Fenton reaction and precipitates as FE(OH)_3_, which leads to emitter clogging (Barakat, 2011; Pitts et al., 2015). Despite this, PAW has the potential to reduce salinity stress in crops (Bafoil et al., 2019; Adhikari et al., 2020). High-iron groundwater should be pretreated prior to PAW generation to ensure chemical stability and prevent irrigation system clogging. Aeration followed by sand or granular filtration is the most common approach, oxidizing Fe²^+^ to Fe³^+^ and removing precipitates (Sharma et al., 2005; Cheng et al., 2020). Biological iron removal via iron-oxidizing bacteria offers an efficient, low-cost alternative (Sharma et al., 2005). For elevated Fe or Fe–Mn mixtures, manganese-oxide-coated dual-media filters enhance removal (Shrestha et al., 2024), while membrane processes (ultrafiltration, nanofiltration, and reverse osmosis) provide high efficiency at greater cost (Khatri et al., 2017). Thus, low-salinity, low-iron water (e.g., rainwater and RO-treated water) should be used for PAW generation, with pretreatment and monitoring of by-products essential for safe agricultural use.

**Supplementary Figure 3b** shows the schematic representation of water activation via plasma discharge and its downstream agricultural applications. Exposure of water to non-TP generates a suite of RONS, including hydrogen peroxide (H_2_O_2_), nitrate (NO_3_^-^), nitrite (NO_2_^-^), hydroxyl radicals (OH), and superoxide anions (O_2_^-^). These species induce significant alterations in the physicochemical properties of water, characterized by acidification (decreased pH), elevated EC, and increased ORP. The modified water exhibits enhanced antimicrobial efficacy, nutrient bioavailability, and redox activity. In agricultural contexts, PAW has demonstrated efficacy in promoting seed germination and early seedling establishment, stimulating vegetative growth and chlorophyll synthesis, augmenting biochemical and antioxidant responses, and mitigating plant disease severity. The schematic underscores the integration of PAW chemical reactivity with biological outcomes, positioning PAW as a promising and sustainable input for crop production through irrigation and plant health management.

Despite its promising agronomic benefits, the direct implementation of PAW in irrigation systems faces several critical challenges. One major concern is the pronounced acidification of PAW, often resulting in pH values below 4, which may adversely affect pH-sensitive crops, disrupt soil buffering capacity, and impair beneficial microbial communities. Low PAW pH can be advantageous depending on specific soil and crop types. In alkaline or calcareous soils, acidified PAW improves micronutrient solubility (e.g., Fe, Zn, and Mn), helping crops like grapevine or potato overcome iron-deficiency chlorosis (Marchner, 2012), and in hydroponic systems, it suppresses pathogens such as *Fusarium* and *Pythium* (Ma et al., 2015). Acidic PAW has also enhanced seed germination and early vigor in wheat and maize under salt stress through reactive species signaling (Bafoil et al., 2019; Adhikari et al., 2020). However, continuous use on acid-sensitive crops (e.g., alfalfa and barley) or already acidic soils risks root injury, nutrient leaching, and Al/Mn toxicity. Thus, while crops like blueberry, grapevine, and lettuce benefit from PAW’s acidity, legumes and cereals on low-pH soils may be disadvantaged. Additionally, the transient nature of short-lived reactive species limits the stability and efficacy of PAW during storage and distribution, while complex soil–water interactions can attenuate reactive species or unpredictably alter nutrient availability and redox dynamics.

In addition to batch plasma–liquid reactors, the interaction of plasma and liquid is known as plasma-aerosol (spray) configuration, which is a promising alternative for high-throughput plasma generation. During this biphasic system, water in mist or spray forms is exposed to RONS effluents, which enhance gas–liquid interfacial area and reactive species mass transfer (Machala et al., 2013; Stancampiano et al., 2019). These setups integrate direct and indirect treatment features producing plasma-treated droplets containing RONS and avoid direct electrode immersion or bulk discharge contact with liquid under treatment. This plasma–aerosol concept has been considered as scalable plasma–liquid platform with distinct advantages in process tuning and throughput offering avenues for agricultural PAW production and field evaluation.

Scaling plasma generation technologies for field-level irrigation presents further technical hurdles, particularly in achieving high-throughput PAW production without compromising energy efficiency. Moreover, the lack of standardized protocols for plasma generation encompassing device architecture, feed gas composition, and discharge parameters introduces variability in PAW chemistry and its biological effects, complicating reproducibility and broader adoption. Addressing these limitations is essential for the reliable and sustainable integration of PAW into agricultural water management practices.

While PAW offers unique advantages for crop production, its direct use in irrigation is limited by strong acidification, variability in reactive species, and short-lived radicals. Several strategies can overcome these challenges, including buffering or neutralization to stabilize pH, dilution with conventional water to reduce acidity while retaining bioactivity, and electrochemical or catalytic post-treatment to adjust nitrate–nitrite ratios and stabilize hydrogen peroxide. Further refinement can be achieved by optimizing plasma discharge parameters and employing on-site generation systems to minimize species decay. Together, these approaches enable tailoring of PAW chemistry to crop and soil requirements, supporting its integration into sustainable irrigation systems. Some approaches to modify PAW chemistry for irrigation and their agronomic implications are listed in **Supplementary Table 3**. PAW chemistry modification involves adjustment of reactive species, pH, and nutrients to enhance its effectiveness in irrigation. The biological effect of PAW remained unclear and can be reproduced by synthetic acidic solutions containing comparable nutrients. The data on these studies were not enough for meta-analysis. Therefore, exploring the contribution of short-lived RONS and detailed analysis are essential. In addition to technical performance, a recent review stated that economic and scalability metrics including energy yield, treatment cost per unit volume, and treatment capacity are to be considered while adopting non-TP technologies in agriculture. These considerations highlight that plasma-based irrigation or the seed treatment system’s sustainability not only demonstrates biological efficacy but also considers energy efficiency, cost, and operational capacity under field conditions (Bilea et al., 2024).

**Figure 8:**
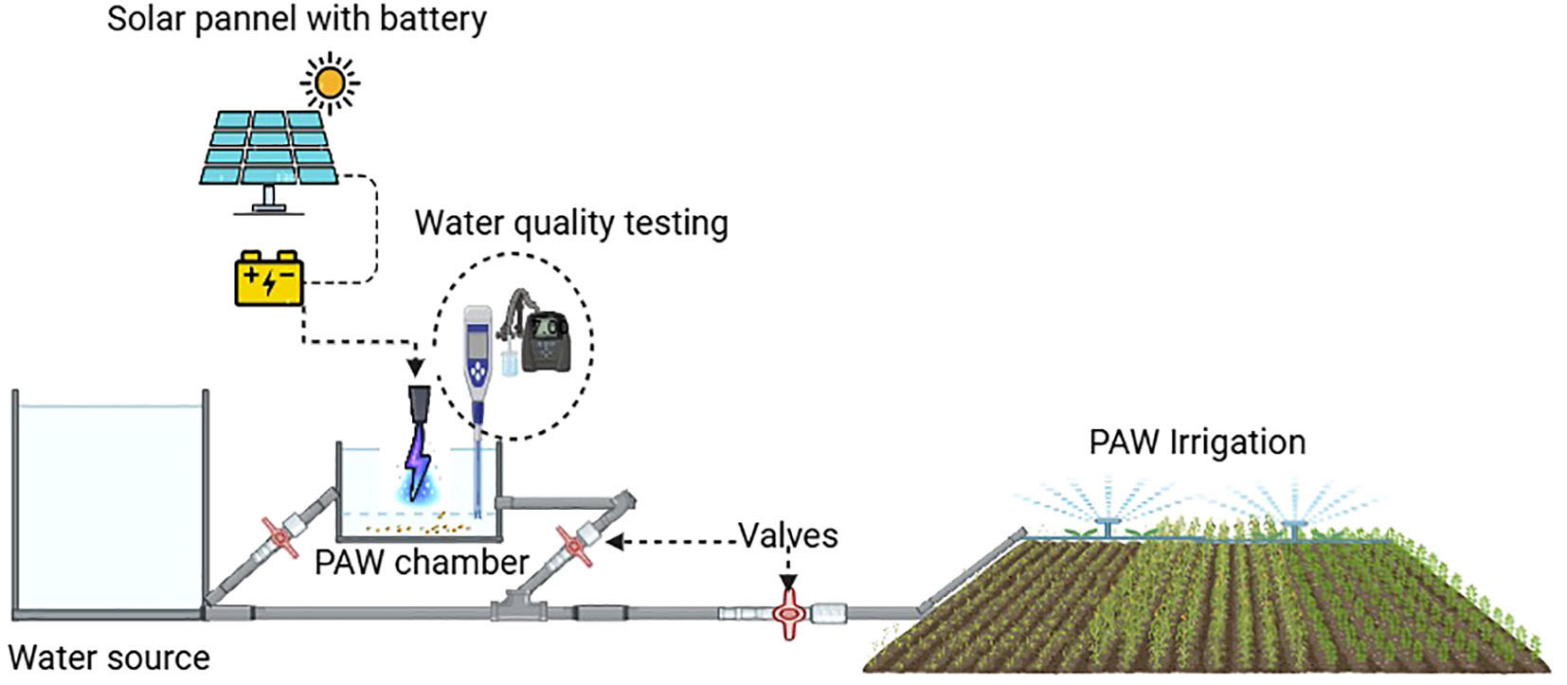
Schematic diagram showing irrigation water source, solar powered plasma water activation, and water quality assessment, controlled by valves before distribution and delivery system to field crops.

Therefore, PAW represents a promising and clear potential to enhance germination, growth, biochemical responses, and disease resistance, while its physiochemical traits favor antimicrobial and nutrient-efficient functions. **Figure 8** illustrates the potential of solar powered irrigation integration with on-site plasma activation chamber where RONS are generated. The inbuilt water quality assessment through embedded sensors will ensure stability and safety for crop application. Valves regulate the flow and pressures through the setup maintaining precise control on water and PAW through an irrigation delivery system. This schematic diagram illustrates the integrated approach of leveraging renewable energy, advanced water treatment technologies, and precision-controlled irrigation systems to promote sustainable agricultural practices. This scalable design regarding solar powered irrigation system with onsite PAW activation chamber and water monitoring sensors demonstrates the practical pathways to scale PAW use for precision irrigation and nutrient management in agriculture. Regarding energy resources, most of the current plasma generation systems rely on grid electricity; future utilization of renewable energies such as solar, wind, and hydropower is cost competitive; and reducing carbon emission is a means for economic feasibility and sustainable PAW production.

## 6 Conclusion

This meta-analysis provides comprehensive evidence supporting PAW potential as a multifunctional input in sustainable agriculture. Across the wide range of studies, PAW application enhanced germination, root and shoot biomass, plant height, stem diameter, and leaf area, which signifies its role in plant establishment and vegetative growth. PAW application significantly increased chlorophyll a, b, c, and carotenoid content in plant leaves. Biochemical traits including TSS, TSP, APX, SOD, CAT, and TPC were consistently increased, supporting stronger metabolic capacity and stress tolerance.

Furthermore, PAW application reduced disease incidence and severity in plants, ensuring better plant health and disease resistance. This highlights PAW potential as a non-chemical alternative for plant protection. Across the studies, crop yield improved with PAW application. The physicochemical properties of water showed alteration with plasma activation, characterized by low pH and elevated EC, nitrate, nitrite, hydrogen peroxide, and ORP reflecting the stable presence of RONS in water. Consistent reduction in pH across all studies reflects the acidifying nature of PAW, which improves nutrient solubility and contributes to pathogens suppression.

Modification of PAW chemistry is essential to ensure safe and agronomically effective irrigation use. Dilution or controlled pH adjustment can moderate acidity and preserve long-lived RONS. Enriching ions, controlling reactive species, and applying chelation collectively enable PAW to be tailored for improved nutrient availability, enhanced plant defense responses, and greater overall crop performance. On-site generation ensures delivery of short-lived species at field scale. Together, these strategies make PAW more compatible with crop and soil systems, supporting its integration into sustainable irrigation practices. It is important to mention that plasma reactors are typically optimized for beneficial outcomes. Numerous positive responses reported in literature reflect the optimized operation settings, and its broader application requires standardized protocol and assessment across different plasma configurations.

Beyond individual physiological responses, PAW application includes crop establishment, enhanced productivity, and plant nutrition. These findings highlight PAW as a multifunctional, eco-friendly technology that integrates chemical and biological mechanisms for crop health improvement, resilience, and productivity. The impact of PAW acidity is context dependent, and the acidifying nature of PAW may influence soil microbial dynamics, potentially enhancing nutrient cycling and long-term soil fertility, particularly in alkaline soils and crops preferring growth in low-pH soils. Future research should focus on standardizing PAW generation methods, green nitrogen production, and delivery methods, and scaling up its use under field conditions. Long-term studies are also needed to assess the cumulative effects of PAW on soil microbial communities, nutrient cycling, and crop performance across different agroecosystems.

## References

[B1] Aceto D. Rotondo P. R. Porfido C. Bottiglione B. Paciolla C. Terzano R. . (2024). Assessing plasma activated water irrigation effects on tomato seedlings. Front. Phys. 12. doi: 10.3389/fphy.2024.1399910. PMID: 41909671

[B2] Adhikari B. Adhikari M. Park G. (2020). The effects of plasma on plant growth, development, and sustainability. Appl. Sci. 10, 6045. doi: 10.3390/app10176045. PMID: 41725453

[B3] Andrade P. E. Savi P. J. Almeida F. S. Carciofi B. A. Pace A. Zou Y. . (2025). Plasma-activated water as a sustainable nitrogen source: Supporting the UN Sustainable Development Goals (SDGs) in controlled environment agriculture. Crops 5, 35. doi: 10.3390/crops5030035. PMID: 41725453

[B4] Antoni V. Cortese E. Navazio L. (2025). Plasma‐activated water to foster sustainable agriculture: Evidence and quest for the fundamentals. Plants People Planet. 7 (6), 1596–1603. doi: 10.1002/ppp3.70025. PMID: 41916768

[B5] Fumiaki M. Tomoya A. Tomoaki I. Kenji E. Shin-Ichi a. Kazuhiro N. (2016) Influence of ozone generated by surface barrier discharge on nematode and plant growth. IEEE Transactions on Plasma Science 44, 3071–3076.

[B6] Bafoil M. Le Ru A. Merbahi N. Eichwald O. Dunand C. Yousfi M. (2019). New insights of low-temperature plasma effects on germination of three genotypes of Arabidopsis thaliana seeds under osmotic and saline stresses. Sci. Rep. 9, 8649. doi: 10.1038/s41598-019-44927-4. PMID: 31209339 PMC6572809

[B7] Barakat M. A. (2011). New trends in removing heavy metals from industrial wastewater. Arabian J. Chem. 4, 361–377. doi: 10.1016/j.arabjc.2010.07.019. PMID: 41916819

[B8] Benítez-López A. Alkemade R. Schipper A. M. Ingram D. J. Verweij P. A. Eikelboom J. A. J. . (2017). The impact of hunting on tropical mammal and bird populations. Sci. (1979). 356, 180–183. doi: 10.1126/science.aaj1891. PMID: 28408600

[B9] Bhabani M. G. Shams R. Dash K. K. (2024). Microgreens and novel non-thermal seed germination techniques for sustainable food systems: a review. Food Sci. Biotechnol. 33, 1541–1557. doi: 10.1007/s10068-024-01529-9. PMID: 38623424 PMC11016050

[B10] Bilea F. Garcia-Vaquero M. Magureanu M. Mihaila I. Mildažienė V. Mozetič M. . (2024). Non-thermal plasma as environmentally-friendly technology for agriculture: A review and roadmap. CRC. Crit. Rev. Plant Sci. 43, 428–486. doi: 10.1080/07352689.2024.2410145. PMID: 41909888

[B11] Chalise R. Tamang A. Kattel A. Sharma S. Basnet S. Khanal R. (2024). Impact of plasma-activated water on germination, growth, and production of green leafy vegetables. AIP Adv. 14, 065318. doi: 10.1063/5.0205372. PMID: 41845761

[B12] Cheng L.-H. Xiong Z.-Z. Cai S. Li D.-W. Xu X.-H. (2020). Aeration-manganese sand filter-ultrafiltration to remove iron and manganese from water: Oxidation effect and fouling behavior of manganese sand coated film. J. Water Process Eng. 38, 101621. doi: 10.1016/j.jwpe.2020.101621. PMID: 41916819

[B13] Chung T.-H. Stancampiano A. Sklias K. Gazeli K. André F. Dozias S. . (2020). Cell electropermeabilisation enhancement by non-thermal-plasma-treated PBS. Cancers (Basel). 12, 219. doi: 10.3390/cancers12010219. PMID: 31963132 PMC7017069

[B14] Fernandes F. A. N. Rodrigues S. (2025). Cold plasma technology for sustainable food production: meeting the United Nations sustainable development goals. Sustain. Food Technol. 3, 32–53. doi: 10.1039/D4FB00209A. PMID: 41909801

[B15] Gao Y. Sha R. Wei G. Yang L. Zhu Y. Liu Y. . (2025). Postharvest application of plasma-activated water delays anthracnose symptom development in apples. Postharvest Biol. Technol. 230, 113760. doi: 10.1016/j.postharvbio.2025.113760. PMID: 41916819

[B16] Graves D. B. Bakken L. B. Jensen M. B. Ingels R. (2019). Plasma activated organic fertilizer. Plasma Chem. Plasma Process. 39, 1–19. doi: 10.1007/s11090-018-9944-9. PMID: 41913934

[B17] Guo D. Liu H. Zhang X. Xiong C. (2024). Plasma activated‐water stimulates aged pepper seeds and promotes seedling growth. Plasma Processes Polym. 21 (5), 2300173. doi: 10.1002/ppap.202300173. PMID: 41916768

[B18] Guo D. Liu H. Zhou L. Xie J. He C. (2021). Plasma‐activated water production and its application in agriculture. J. Sci. Food Agric. 101, 4891–4899. doi: 10.1002/jsfa.11258. PMID: 33860533

[B19] Jirešová J. Scholtz V. Julák J. Šerá B. (2022). Comparison of the effect of plasma-activated water and artificially prepared plasma-activated water on wheat grain properties. Plants 11, 1471. doi: 10.3390/plants11111471. PMID: 35684244 PMC9183031

[B20] Kaneko T. Sasaki S. Hokari Y. Horiuchi S. Honda R. Kanzaki M. (2015). Improvement of cell membrane permeability using a cell-solution electrode for generating atmospheric-pressure plasma. Biointerphases 10, 029521. doi: 10.1116/1.4921278. PMID: 25997854

[B21] Karimi J. Bansal S. A. Kumar V. Pasalari H. Badr A. A. Nejad Z. J. (2024). Effect of cold plasma on plant physiological and biochemical processes: A review. Biol. (Bratisl). 79, 3475–3487. doi: 10.1007/s11756-024-01794-3. PMID: 41913934

[B22] Khatri N. Tyagi S. Rawtani D. (2017). Recent strategies for the removal of iron from water: A review. J. Water Process Eng. 19, 291–304. doi: 10.1016/j.jwpe.2017.08.015. PMID: 41916819

[B23] Kooshki S. Pareek P. Janda M. Machala Z. (2024). Selective reactive oxygen and nitrogen species production in plasma-activated water via dielectric barrier discharge reactor: An innovative method for tuning and its impact on dye degradation. J. Water Process Eng. 63, 105477. doi: 10.1016/j.jwpe.2024.105477. PMID: 41916819

[B24] Kumar R. Thakur K. A. Vikram A. Vaid A. Rane R. (2018). Effect of plasma treatment on seed crop characters of okra [Abelmoschus esculentus (L.) under field conditions. Int. J. Curr. Microbiol. Appl. Sci. 7, 967–976. doi: 10.20546/ijcmas.2018.712.120

[B25] Kuzin A. I. Kashirskaya N. Y. Solovchenko A. E. Kochkina A. M. Stepantsowa L. V. Krasin V. N. . (2024). Influence of plasma-activated water on foliar and fruit micronutrient content and plant protection efficiency. Horticulturae 10, 55. doi: 10.3390/horticulturae10010055. PMID: 41725453

[B26] Kuzin A. Solovchenko A. Khort D. Filippov R. Lukanin V. Lukina N. . (2023). Effects of plasma-activated water on leaf and fruit biochemical composition and scion growth in apple. Plants 12, 385. doi: 10.3390/plants12020385. PMID: 36679098 PMC9865715

[B27] Li L. Zhang L. Dong Y. (2025). Seed priming with cold plasma mitigated the negative influence of drought stress on growth and yield of rapeseed (Brassica napus L.). Ind. Crops Prod. 228, 120899. doi: 10.1016/j.indcrop.2025.120899. PMID: 41916819

[B28] Liu H. Zhang X. Cui Z. Ding Y. Zhou L. Zhao X. (2022). Cold plasma effects on the nutrients and microbiological quality of sprouts. Food Res. Int. 159, 111655. doi: 10.1016/j.foodres.2022.111655. PMID: 35940774

[B29] Lo Porto C. Ziuzina D. Los A. Boehm D. Palumbo F. Favia P. . (2018). Plasma activated water and airborne ultrasound treatments for enhanced germination and growth of soybean. Innovative Food. Sci. Emerging Technol. 49, 13–19. doi: 10.1016/j.ifset.2018.07.013. PMID: 41916819

[B30] Lukes P. Dolezalova E. Sisrova I. Clupek M. (2014). Aqueous-phase chemistry and bactericidal effects from an air discharge plasma in contact with water: evidence for the formation of peroxynitrite through a pseudo-second-order post-discharge reaction of H _2_ O _2_ and HNO _2_. Plasma Sources Sci. Technol. 23, 15019. doi: 10.1088/0963-0252/23/1/015019

[B31] Ma R. Wang G. Tian Y. Wang K. Zhang J. Fang J. (2015). Non-thermal plasma-activated water inactivation of food-borne pathogen on fresh produce. J. Hazard. Mater. 300, 643–651. doi: 10.1016/j.jhazmat.2015.07.061. PMID: 26282219

[B32] Machala Z. Tarabova B. Hensel K. Spetlikova E. Sikurova L. Lukes P. (2013). Formation of ROS and RNS in water electro-sprayed through transient spark discharge in air and their bactericidal effects. Plasma Processes Polym. 10, 649–659. doi: 10.1002/ppap.201200113. PMID: 41916768

[B33] Malahlela H. K. Belay Z. A. Mphahlele R. R. Sigge G. O. Caleb O. J. (2024). Recent advances in activated water systems for the postharvest management of quality and safety of fresh fruits and vegetables. Compr. Rev. Food Sci. Food Saf. 23 (2), e13317. doi: 10.1111/1541-4337.13317. PMID: 38477217

[B34] Marchner (2012). Marschner’s mineral nutrition of higher plants (The Univ. of Adelaide, Australia: Academic press). doi: 10.1016/C2009-0-63043-9, PMID:

[B35] Meropoulis S. Aggelopoulos C. A. (2023). Plasma microbubbles vs gas-liquid DBD energized by low-frequency high voltage nanopulses for pollutants degradation in water: Destruction mechanisms, composition of plasma-activated water and energy assessment. J. Environ. Chem. Eng. 11, 109855. doi: 10.1016/j.jece.2023.109855. PMID: 41916819

[B36] Misra N. N. Naladala T. Alzahrani K. J. Sreelakshmi V. P. Negi P. S. (2024). Design of a continuous plasma activated water (PAW) disinfection system for fresh produce industry. Innovative Food. Sci. Emerging Technol. 97, 103845. doi: 10.1016/j.ifset.2024.103845. PMID: 41916819

[B37] Motulsky H. (2007) Prism 5 statistics guide. GraphPad Software 31, 39–42.

[B38] Mubeen M. Jatoi W. N. Hashmi M. Z. Ahmad M. (2025). Innovations in agricultural water management. Eds. Mubeen M. Jatoi W. N. Hashmi M. Z. Ahmad M. (Cham: Springer Nature Switzerland). doi: 10.1007/978-3-031-91883-4, PMID:

[B39] Mumtaz S. Khan R. Rana J. N. Javed R. Iqbal M. Choi E. H. . (2023). Review on the biomedical and environmental applications of nonthermal plasma. Catalysts 13, 685. doi: 10.3390/catal13040685. PMID: 41725453

[B40] Obradović B. M. Ivković S. S. Kuraica M. M. (2008). Spectroscopic measurement of electric field in dielectric barrier discharge in helium. Appl. Phys. Lett. 92, 191501. doi: 10.1063/1.2927477. PMID: 41411595

[B41] Panngom K. Lee S. H. Park D. H. Sim G. B. Kim Y. H. Uhm H. S. . (2014). Non-thermal plasma treatment diminishes fungal viability and up-regulates resistance genes in a plant host. PloS One 9, e99300. doi: 10.1371/journal.pone.0099300. PMID: 24911947 PMC4049833

[B42] Pérez-Pizá M. C. Prevosto L. Grijalba P. E. Zilli C. G. Cejas E. Mancinelli B. . (2019). Improvement of growth and yield of soybean plants through the application of non-thermal plasmas to seeds with different health status. Heliyon 5, e01495. doi: 10.1016/j.heliyon.2019.e01495. PMID: 31011650 PMC6462543

[B43] Pitts D. Haman D. Smajstrla A. (2015) Causes and prevention of emitter clogging in microirrigation systems. https://edis.ifas.ufl.edu/publication/AE032.

[B44] Priatama R. A. Beak H. K. Park S. Song I. Park S. J. Kim S. B. . (2025). Tomato yield enhancement with plasma-activated water as an alternative nitrogen source. BMC Plant Biol. 25, 668. doi: 10.1186/s12870-025-06701-9. PMID: 40394503 PMC12090648

[B45] Puač N. Škoro N. (2025). Plasma–liquid interaction for agriculture—a focused review. Plasma Processes Polym. 22 (1), 2400208. doi: 10.1002/ppap.202400208. PMID: 41916768

[B46] Rashid M. Rashid M. M. Alam M. S. Talukder M. R. (2022). Stimulating effects of plasma activated water on growth, biochemical activity, nutritional composition and yield of potato (Solanum tuberosum L.). Plasma Chem. Plasma Process. 42, 131–145. doi: 10.1007/s11090-021-10216-0. PMID: 41913934

[B47] Rashid M. Rashid M. M. Reza M. A. Talukder M. R. (2021). Combined effects of air plasma seed treatment and foliar application of plasma activated water on enhanced paddy plant growth and yield. Plasma Chem. Plasma Process. 41, 1081–1099. doi: 10.1007/s11090-021-10179-2. PMID: 41913934

[B48] Rathore V. Nema S. K. (2025). A nitrogen alternative: Use of plasma activated water as nitrogen source in hydroponic solution for radish growth. Plasma Chem. Plasma Process. 45, 1103–1123. doi: 10.1007/s11090-025-10569-w. PMID: 41913934

[B49] Robert E. Akishev Y. Bauzin J.-G. Collet G. Dozias S. Ermolaeva S. A. . (2025). State of the art on the understanding of plasma jet interaction with skin tissue models. Plasma Med. 15, 53–77. doi: 10.1615/PlasmaMed.2025060231. PMID: 36976008

[B50] Robert E. Darny T. Dozias S. Iseni S. Pouvesle J. M. (2015). New insights on the propagation of pulsed atmospheric plasma streams: From single jet to multi jet arrays. Phys. Plasmas 22, 122007. doi: 10.1063/1.4934655. PMID: 41411595

[B51] Scanlon B. R. Fakhreddine S. Rateb A. de Graaf I. Famiglietti J. Gleeson T. . (2023). Global water resources and the role of groundwater in a resilient water future. Nat. Rev. Earth Environ. 4, 87–101. doi: 10.1038/s43017-022-00378-6. PMID: 41896565

[B52] Scholtz V. Jirešová J. Lokajová E. Měřínská T. Thonová L. Šerá B. (2025). Is plasma activated water really magical? A reflection on the phenomenon. Plasma Chem. Plasma Process. 45, 1337–1351. doi: 10.1007/s11090-025-10565-0. PMID: 41913934

[B53] Seleiman M. F. Ali N. Nungula E. Z. Gitari H. I. Alhammad B. A. Battaglia M. L. (2024). Enhancing germination and seedling growth of barley using plasma-activated water (PAW) with neutralized pH. Cogent Food Agric. 10, 2390162. doi: 10.1080/23311932.2024.2390162. PMID: 41909888

[B54] Šerá B. Scholtz V. Jirešová J. Khun J. Julák J. Šerý M. (2021). Effects of non-thermal plasma treatment on seed germination and early growth of leguminous plants—a review. Plants 10, 1616. doi: 10.3390/plants10081616. PMID: 34451662 PMC8401949

[B55] Shaji M. Rabinovich A. Surace M. Sales C. Fridman A. (2023). Physical properties of plasma-activated water. Plasma 6, 45–57. doi: 10.3390/plasma6010005. PMID: 41725453

[B56] Sharma S. K. Petrusevski B. Schippers J. C. (2005). Biological iron removal from groundwater: a review. J. Water Supply: Res. Technology-Aqua 54, 239–247. doi: 10.2166/aqua.2005.0022. PMID: 41910062

[B57] Shrestha A. M. Kazama S. Sawangjang B. Takizawa S. (2024). Improvement of removal rates for iron and manganese in groundwater using dual-media filters filled with manganese-oxide-coated sand and ceramic in Nepal. Water (Basel). 16, 2450. doi: 10.3390/w16172450. PMID: 41725453

[B58] Siddique S. S. Hardy G. E. S. J. Bayliss K. L. (2018). Cold plasma: a potential new method to manage postharvest diseases caused by fungal plant pathogens. Plant Pathol. 67, 1011–1021. doi: 10.1111/ppa.12825. PMID: 41875165

[B59] Silverj R. Di Palma L. Ermini A. Macchia A. (2025). Toward sustainable chlorine dioxide: Managing and minimizing by‐product generation. doi: 10.20944/preprints202505.1968.v1, PMID: 41116858

[B60] Sivachandiran L. Khacef A. (2017). Enhanced seed germination and plant growth by atmospheric pressure cold air plasma: combined effect of seed and water treatment. RSC Adv. 7, 1822–1832. doi: 10.1039/C6RA24762H. PMID: 41909801

[B61] Song I. Jeon H. Priatama R. A. Gayathri S. Ko K. Lee Y. K. (2023). Effect of plasma-activated water on peanut seed germination and vegetative growth in a hydroponic system. Plant Biotechnol. Rep. 17, 573–583. doi: 10.1007/s11816-023-00847-4. PMID: 41913934

[B62] Stancampiano A. Gallingani T. Gherardi M. Machala Z. Maguire P. Colombo V. . (2019). Plasma and aerosols: Challenges, opportunities and perspectives. Appl. Sci. 9, 3861. doi: 10.3390/app9183861. PMID: 41725453

[B63] Thirumdas R. Kothakota A. Annapure U. Siliveru K. Blundell R. Gatt R. . (2018). Plasma activated water (PAW): Chemistry, physico-chemical properties, applications in food and agriculture. Trends Food Sci. Technol. 77, 21–31. doi: 10.1016/j.tifs.2018.05.007. PMID: 41916819

[B64] Veerana M. Mumtaz S. Rana J. N. Javed R. Panngom K. Ahmed B. . (2024). Recent advances in non-thermal plasma for seed germination, plant growth, and secondary metabolite synthesis: A promising frontier for sustainable agriculture. Plasma Chem. Plasma Process. 44, 2263–2302. doi: 10.1007/s11090-024-10510-7. PMID: 41913934

[B65] Viechtbauer W. (2010). Conducting meta-analyses in R with the metafor package. J. Stat. Software 36, 1–48. doi: 10.18637/jss.v036.i03

[B66] Vijayarangan V. Maaroufi Z. Rouillard A. Gaetan-Zin S. Dozias S. Escot-Bocanegra P. . (2026). Plasma jet and plasma treated aerosol induced permeation of reconstructed human epidermis. Bioelectrochemistry 167, 109060. doi: 10.1016/j.bioelechem.2025.109060. PMID: 40774184

[B67] Waghmare R. (2021). Cold plasma technology for fruit based beverages: A review. Trends Food Sci. Technol. 114, 60–69. doi: 10.1016/j.tifs.2021.05.018. PMID: 41916819

[B68] Wong K. S. Chew N. S. L. Low M. Tan M. K. (2023). Plasma-activated water: Physicochemical properties, generation techniques, and applications. Processes 11, 2213. doi: 10.3390/pr11072213. PMID: 41725453

[B69] Yasir M. Hossain A. Pratap-Singh A. (2025). Pesticide degradation: Impacts on soil fertility and nutrient cycling. Environments 12, 272. doi: 10.3390/environments12080272. PMID: 41725453

