# Supplementary material for: Global meta-analysis of plasma-activated water for improving crop establishment, productivity, and health
Source: Front Plant Sci. 2026 Apr 15;17:1754073. doi: 10.3389/fpls.2026.1754073 (PMC13124626; doi:10.3389/fpls.2026.1754073)
Supplement: Supplementary file 1 [file DataSheet1.docx]

Supplementary Material

# Supplementary figures

**(b)**

**(a)**

Supplementary Figure 1. Number of published papers on PAW for irrigation by country, reflecting geographical distribution (a) and annual distribution of published research papers till January-2025, reflecting publications per year over time (b)

**(b)**


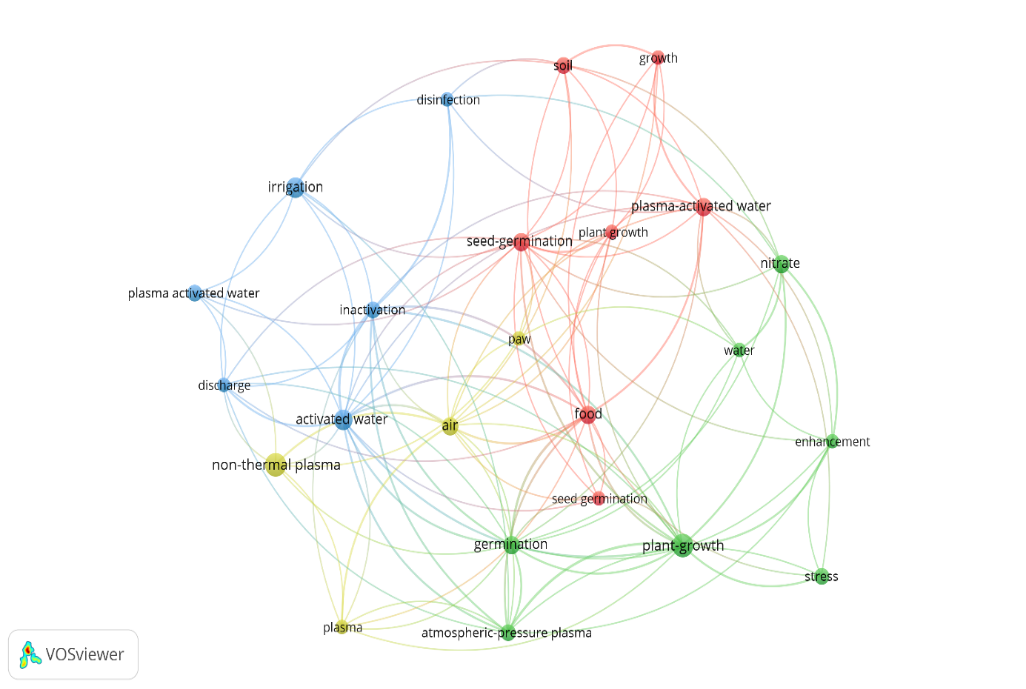


**(a)**

Supplementary Figure 2. (a) Keywords co-occurrence network visualization generated through VOSviewer showing the linkage and clustering within PAW for irrigation research domain and (b) Number of studies conducted on plasma generation methods and reactor types such as Dielectric Barrier Discharge Plasma Systems (DBDPS), Plasma Jet Systems (PJS), Gliding Arc Discharge Plasma Systems (GADPS), Glow Discharge Plasma Systems (GDPS), Transient Spark & Spark Discharge Plasma Systems (TS & SDPS), Arc Discharge Plasma Systems (ADPS), Atmospheric Pressure & Hybrid Plasma Systems (AP & HPS), Electrochemical Plasma Systems (EPS) and Pulse & Reactor-Based Plasma Systems (P& RBPS) used for irrigation


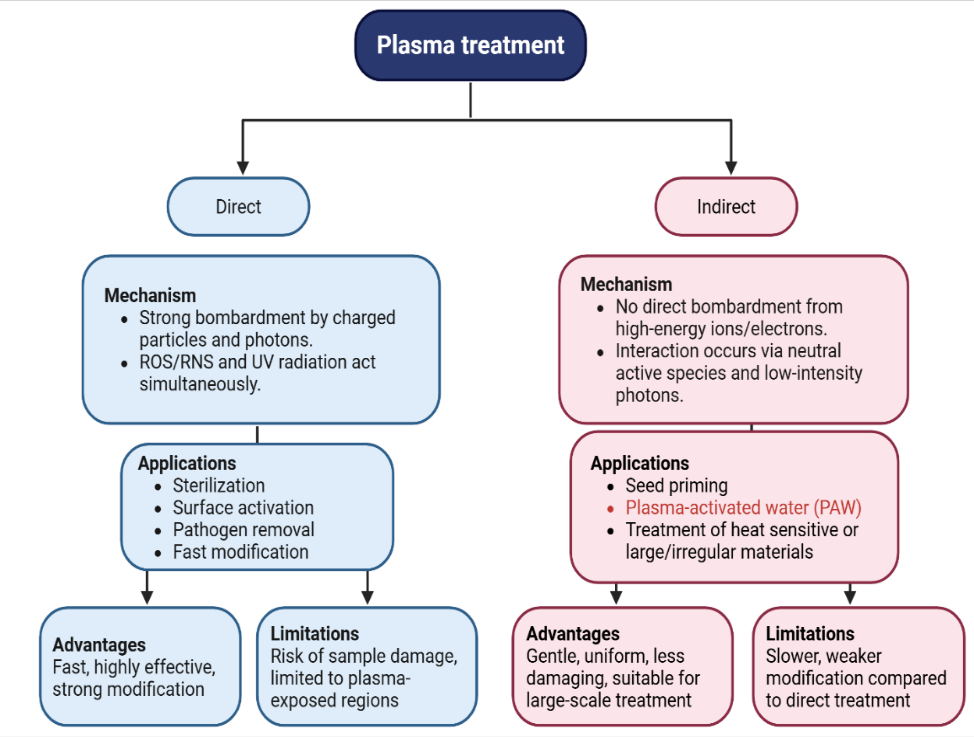

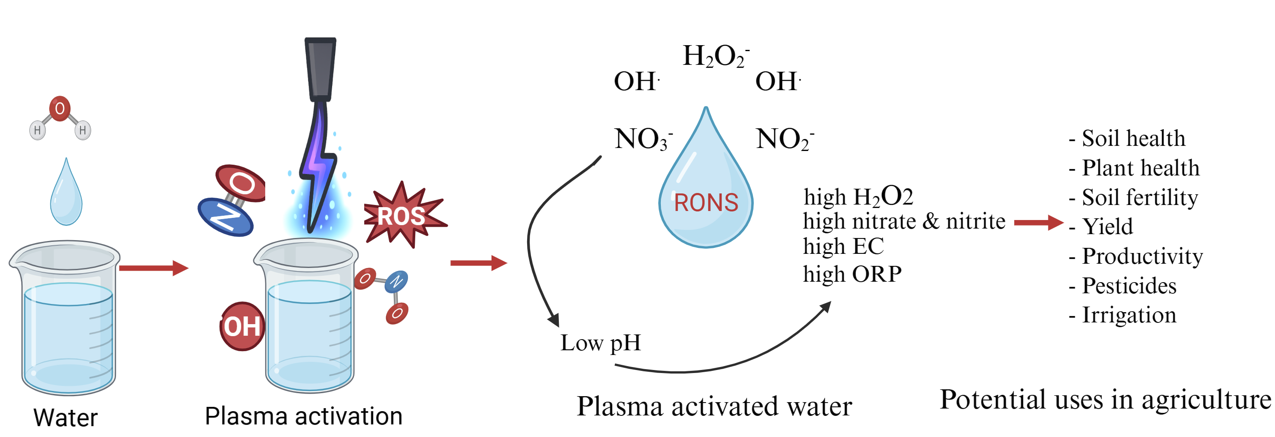


**(b)**

**(a)**

Supplementary Figure 3. (a) Mechanisms, applications, advantages and limitations of plasma direct and indirect treatments and (b) mechanism of water activation through plasma, reactive species, properties after activation and its potential uses in agriculture

**2 Supplementary Tables**

Supplementary Table 1. Summary of apparatus types and operational parameters (voltage, frequency, and treatment time) used for plasma-activated water generation.

| **Method** | **Making method/ apparatus /Reactor type** | **Voltage** | **Frequency** | **Time** |
| --- | --- | --- | --- | --- |
| Arc Discharge Plasma Systems | PAW was prepared by arc discharge O2 and Ar plasmas | 3-6 kV | 3–10kHz | 10 min |
|  | Three-arc plasma-chemical unit | 15 kV | 100 Hz |  |
|  | Plasma arc discharge |  |  |  |
|  | High-voltage electrical discharge (HVED) device | 30 kV | 50 Hz | 30 s |
|  | Plasma arc discharge | 10 kV | 20 KHz |  |
|  | Electric arc discharge plasma | 10.61 kV | 50 Hz |  |
|  | Arc discharge plasma | 6-10 kV | 40 KHz |  |
| Atmospheric Pressure & Hybrid Plasma Systems | Cold plasma (CP) generation system |  | 18 kHz | 30 min |
|  | Low frequency microwave hybrid plasma (LFMHP) and low-Frequency plasma jet (LFPJ) | 7 kV | 10 kHz |  |
|  | Atmospheric pressure multi-tube air bubble discharge | 10 kV | 0.05–5  kHz |  |
|  | bubble spark discharge (BSD) | 150 V |  |  |
|  | Atmospheric pressure plasma system (DE-21436, Marschacht, Germany) |  |  |  |
|  | Non-thermal plasma (NTP) technology | 347 kV | 10 kHz |  |
|  | T-shaped point-to-point NTP reactor |  | 250 Hz |  |
|  | Atmospheric low temperature isoelectric test power |  |  | 30-60 min |
|  | Microwave torch plasma system |  |  |  |
| Dielectric Barrier Discharge (DBD) Plasma Systems | Plasma Device (MECDBD) | 3 kV | 20 kHz | 1 hour |
|  | Surface dielectric barrier discharge (DBD) | 10 kV | 6.2 kHz | 5-20 min |
|  | Dielectric barrier discharge (DBD) | 11.8 kV | 50 Hz | 1-20 minutes |
|  | Dielectric barrier discharge (DBD) | 16 kV | 11 kHz | 15 second |
|  | Dielectric barrier discharge | 19 kV | 1 kHz | 10 minutes |
|  | Delectric barrier discharges (DBD) |  |  | 2.5–15 min |
|  | Dielectric barrier discharge (DBD) | 12 kV | 6 kHz |  |
|  | Dielectric barrier discharge diffusor (DBDD) system | 8 kV | 60 kHz | 2 min |
|  | Dielectric barrier discharge (DBD) | 24 kV | 1,5 kHz | 5-30 min |
|  | Surface dielectric barrier discharge (SDBD) device | 8 kVpp | 17 kHz |  |
|  | Dielectric Barrier Discharge (DBD) system | 80 kV | 50 Hz | 1-5 min |
|  | surface dielectric barrier discharge (SDBD) | 8 kV | 14.4 kHz | 20-60 min |
|  | Surface dielectric barrier discharge (SDBD) | 8 kV | 17 kHz |  |
|  | Surface dielectric barrier discharge (SDBD) |  | 17 kHz | 5-40 min |
|  | Diffuse Coplanar Surface Barrier Discharge (DCSBD) plasma system | 20 kV | 15 kHz |  |
|  | Dielectric barrier discharge (DBD) | 160 kV | 145 Hz | 5 min |
|  | Dielectric barrier discharge (DBD) plasma system |  | 10 kHz |  |
|  | Dielectric barrier discharge (DBD) plasma system | 0-50 kV | 50 Hz |  |
|  | Low pressure dielectric barrier air discharge plasma | 1–10 kV | 1–8 kHz |  |
|  | Low-pressure dielectric barrier discharge (LPDBD) plasma | 5–10 kV | 3–8 kHz | 1-4 min |
|  | Dielectric barrier discharge (DBD) | 11.7 kV | 50 Hz | 4 min |
|  | Dielectric barrier discharge plasma-activated mist (PAM) | 20 kV |  | 20 min |
|  | Dielectric barrier discharge (DBD) | 10 kV |  | 10 min |
|  | Dielectric barrier discharge (DBD) plasma | 24 kV | 1.5 kHz | 30-45 min |
|  | Dielectric barrier discharge (DBD) plasma device | 2-50 kV | 10 kHz | 15-25 min |
|  | Surface Dielectric Barrier Discharge | 8 kV | 10 kHz | 60-80 s |
|  | Dielectric barrier discharge (DBD) plasma | 30 kV |  | 30 min |
|  | Dielectric barrier discharge (DBD) plasma | 6 kVp-p | 18 kHz | 0-30 min |
|  | Dielectric barrier discharge | 16 kV | 11 kHz | 2-10 min |
|  | Dielectric barrier discharge plasma (DBD) | 3.6 kV | 7.5 Hz |  |
|  | Dielectric barrier discharge plasma (DBD) | 40 V | 10 kHz |  |
|  | ME-CDBD plasma source |  |  |  |
|  | Dielectric barrier discharge plasma (DBD) |  |  |  |
|  | DBD plasma | 37 kV, 43 kV, or 49 kV | 15.5 kHz |  |
|  | Dielectric barrier discharge plasma |  | 120 MHz |  |
|  | Dielectric barrier discharge plasma | 19-30 kV |  |  |
|  | Double dielectric barrier discharge reactors (DBD | 40 kV | 1 KHz | 30 min |
|  | Dielectric Barrier Discharge Cold Plasma System | 7 kV | 25 KHz | 15 min |
|  | Dielectric Barrier Discharge Cold Plasma System | 10 kV |  |  |
|  | Dielectric barrier discharge (DBD) | 6 kV | 21.5 kHz |  |
|  | Cylindrical double DBD (D-DBD) | 20 kV | 50 Hz | 20 min |
|  | Dielectric barrier discharge (DBD) |  |  |  |
|  | Dielectric barrier device (DBD) | 16 kV | 50 Hz |  |
|  | Dielectric barrier discharge plasma device, Dielectric barrier Discharge pencil plasma jet | 10 kV | 40 kHz |  |
|  | Corona dielectric barrier discharge (cDBD) | 17-27 kV | 0.3 Hz | 5-15 min |
|  | Dielectric Barrier Discharge (DBD) | 0-20kV | 9 kHz | 10 min |
| Electrochemical Plasma Systems | Electrochemical setup containing an electrolyte vessel fitted with active (platinum (Pt)) and neutral (Pt) electrodes | 300 V | 440 kHz | 6 hour |
|  | PAW 1 (using an electrochemical unit electrolyte vessel) PAW2 (argon plasma jet) | | 440 kHz | 6 hour |
|  | Gas plasma-activated water (GPAW) | 15.8 kV | 27.1 kHz |  |
|  | electric discharge |  |  |  |
| Gliding Arc Discharge (GAD) Plasma Systems | Three-arc plasma-chemical unit | 15 kV | 100 Hz |  |
|  | Atmospheric pressure gliding discharge | 0–15 kV | 50 Hz | 5-15 min |
|  | Gliding Arc |  |  | 30 min |
|  | Gliding arc plasma | 10–24 kV | 6 kHz | 90 min |
|  | Gliding arc discharge (GAD) | 9 kV | 50 Hz | 5 and 10 min |
|  | Gliding Arc discharge | 9.7 kV, | 50 Hz |  |
|  | Gliding arc discharge | 3.6 kV |  |  |
|  | Gliding arc discharge | 9.19 kV | 50 Hz |  |
|  | Gliding arc plasma discharge |  |  |  |
|  | Gliding arc discharge plasma | 10 kV | 20 kHz |  |
|  | Gliding arc discharge | 15 kV |  |  |
|  | Gliding arc discharge | 1.52 kV |  | 4-16 min |
|  | Air gliding arc (GA) plasma | 6-10 kV | 700-900 kHz | 2-6 min |
|  | Gliding arc discharge (GAD) | 13.30 kV |  | 5 or 10 min |
|  | Gliding arc discharge (GAD) | 10 kVp | 10 kHz |  |
|  | GlidArc discharge method | 7 kV | 1 kHz | 30 min |
|  | GlidArc discharge method |  |  |  |
|  | Gliding arc discharge (GAD) | 3.8 kV | 50 Hz |  |
|  | High Voltage Electrical Discharge (HVED) |  | 30 Hz | 30 s |
|  | Gliding arc plasma | 15 kV | 25 kHz |  |
|  | Gliding Arc Plasma Activated Water (GAPAW) | 9-10 kV | 40 KHz | 10 min |
| Glow Discharge Plasma Systems | Glow discharge | 250–350 V | 0.11 MHz |  |
|  | Low pressure (100 Torr) glow air discharge LPGAD | 1–10 kV | 0.5–10 kHz |  |
|  | Glow discharge |  | 110 kHz |  |
|  | Atmospheric glow discharge plasma | 1-10 kV | 1-10 kHz |  |
|  | Glow Discharge Plasma treatment |  |  |  |
|  | Glow discharge |  |  |  |
|  | Glow discharge plasma | 250-350 V | 0.44 MHz |  |
| Plasma Jet Systems | Plasma jet | 10 kV |  |  |
|  | Plasma jet | 1-10kV | 1-10kHz | 9-12 min |
|  | Pinhole plasma jet |  |  |  |
|  | Plasma jet | 10 kV | 33 kHz |  |
|  | Plasma jet | 12 kV | 28 kHz. | 40 min |
|  | Plasma jet | 1–10 kV | 0.5–10 kHz | 10 min |
|  | Plasma jet |  |  |  |
|  | Plasma jet | 7 kV |  | 1-5 min |
|  | Plasma jet system | 220 V | 13.56 MHz |  |
|  | Plasma jet | 302 V | 23 kHz |  |
|  | Plasma jet |  |  |  |
|  | Plasma jet | 10 kV | 40 kHz |  |
|  | Plasma jet | 0.5-10 kV | 50 Hz |  |
|  | Plasma jet |  |  | 15-60 min |
|  | Plasma jet |  |  |  |
|  | Plasma jet device |  |  | 30min |
|  | Plasma jet | 1–10 kV | 1–10 kHz |  |
|  | Atmospheric-pressure plasma jet (APPJ) | 4 kV |  | 10 min |
|  | Atmospheric plasma jet | 12 kV | 28 kHz | 30 min |
|  | Atmospheric pressure plasma jet (APPJ) | 12 kV | 25 kHz |  |
|  | plasma jet | 2 – 7 kV | 50 kHz |  |
|  | Atmospheric pressure plasma jet (Plasmatreat Inc., IL, USA) | 295 V | 22.5 kHz | 5 min |
|  | Plasma jet | 8.2 kV |  |  |
|  | Atmospheric pressure plasma jet (APPJ) |  |  |  |
|  | Plasma jet device | 10 kV | 10 kHz | 5 min |
|  | Plasma jet | 18 kV | 10 kHz | 1-6 min |
|  | Atmospheric pressure air plasma jet | 35 V | 35 kHz | 5-25 min |
|  | Plasma jet | 10 kV | 25 KHz |  |
|  | Atmospheric pressure plasma jet |  |  |  |
|  | Atmospheric pressure plasma jet | 3 kV | 16 kHz |  |
|  | Atmospheric pressure air plasma jet (APAPJ) |  | 25 KHz |  |
|  | Plasma jet |  |  |  |
|  | Atmospheric pressure plasma jet (APPJ) | 20 kV |  | 10-20 min |
| Pulse & Reactor-Based Plasma Systems | Plasma reactor |  | 13 kHz |  |
|  | Pulse electrical discharge |  | 60 and 100 Hz |  |
|  | Radio frequency discharge device |  | 13.56 MHz | 15 s |
| Transient Spark & Spark Discharge Systems | Transient spark discharge | 11-13 kV | 1.5-3 k Hz | 1 min |
|  | Transient spark (TS) discharge | 20 kV |  | 1 hour |
|  | Transient spark (TS) discharge and glow discharge (GD) | 20 kV |  | 25 min for TS and 2 min for GD |
|  | Transient spark | 9 kV |  | 25 min |
|  | Transient spark discharge | 16 kV | 2–3 kHz |  |
|  | Transient spark discharge | 20 kV |  |  |
|  | Spark plug plasma generator (SPPG) | 9-15 kV | 15 kHz | 30 min |
|  | Transient spark (TS) discharge | 10–13 kV | 2–3 kHz |  |
|  | Transient spark discharge apparatus | 9 kV |  |  |
|  | Transient spark (TS) discharge | 20 kV | 1–3 kHz |  |
|  | Spark discharge plasma | 20 kV | 1 Hz | 5 min |
|  | Transient spark (TS) | 4.6 kV |  | 20 min |

Supplementary Table 2. PAW effectiveness for target pathogen or diseases in different crops

| **Pathogen Type** | **Pathogen (s)** | **Disease / Common Name** | **Host (s) with references** |
| --- | --- | --- | --- |
| Fungi | *Phytophthora infestans* | Late blight | Tomato, Potato (Solanaceae)(Djeugap et al., 2024; Guo et al., 2024) |
|  | *Pyricularia oryzae (syn. Magnaporthe oryzae)* | Rice blast | Rice (Poaceae) (Hasan, 2020) |
|  | *Venturia inaequalis* | Apple scab | Apple (Rosaceae) (Kuzin et al., 2023) |
|  | *Lecanicillium fungicola* | Dry bubble | Button mushroom (Agaricaceae) (Pourbagher et al., 2023) |
|  | *Bipolaris oryzae* | Brown spot of rice | Rice (Poaceae) (Hasan, 2020) |
|  | *Fusarium graminearum* | Fusarium head blight | Wheat, Barley (Poaceae) (Doshi et al., 2024; Feizollahi et al., 2023; Guo et al., 2021; Ju et al., 2023) |
|  | *Fusarium spp.* | Wilt / rot diseases | Sorghum, Wheat, Strawberry, Cotton (various) (Ashurov et al., 2022; Danilejko et al., 2021) |
|  | *Colletotrichum spp.* | Anthracnose | Pepper (Solanaceae) (Ahmad et al., 2022) |
| Bacteria | *Pseudomonas tolaasii* | Brown blotch | Button mushroom (Pourbagher et al., 2023) |
|  | *Xanthomonas vesicatoria* | Bacterial spot | Tomato (Solanaceae) (Bertaccini et al., 2015; Pérez-Pizá et al., 2019) |
|  | *Xanthomonas campestris* | Black rot | Cabbage (Brassicaceae) (Suwannarat et al., 2024) |
|  | *Enterobacter aerogenes* | Contamination | Lettuce, kale, spinach (Brassicaceae, Asteraceae) (Tan and Karwe, 2021) |
|  | *Clavibacter michiganensis subsp. michiganensis* | Bacterial canker | Kiwifruit (Actinidiaceae) (Liu et al., 2024) |
| Viruses | *Tomato mottle mosaic virus (ToMMV)* | ToMMV infection | Tomato (Aceto et al., 2024) |
| Pests | *Tetranychus urticae* | Two‑spotted spider mite | Tomato (Savi et al., 2025) |
| Yeasts | *Saccharomyces cerevisiae* | Contaminant | Wheat, Grapes (Guo et al., 2017; Jirešová et al., 2022) |
| Foodborne / Enteric bacteria | *Escherichia coli, Salmonella enterica (incl. S. Typhimurium), Listeria monocytogenes* | Foodborne contamination | Multiple crops (lettuce, melon, legumes, broccoli, soybean, alfalfa) (Darmanin et al., 2021; Gutiérrez-León et al., 2022; Han et al., 2024; Machado-Moreira et al., 2021; Mošovská et al., 2022; Rothwell et al., 2023; Zhang et al., 2022) |

Supplementary Table 3. Summary of chemical modification approaches for plasma-activated water (PAW) in irrigation. Each strategy is categorized by its underlying approach, mechanism of action, and potential agronomic benefits, highlighting how tailored PAW chemistry can enhance water efficiency, nutrient delivery, and crop performance in scalable agricultural systems.

| **Approach** | **Strategy** | **Mechanism** | **Potential benefits** |
| --- | --- | --- | --- |
| Buffering and neutralization | Addition of bicarbonates, phosphates, organic matter, biochar and lime | Neutralize acidity, stabilize pH | Prevent soil acidity, enhance Ca, K and P uptake, supports microbial activity |
| Dilution | Mixing PAW with irrigation water | Decrease ionic strength and acidity while retains RONS | Safe for sensitive crops, simple integration to irrigation systems, minimize phytotoxicity |
| Electrochemical and catalytic post-treatment | Electrochemical adjustment, catalytic filters | Modifies NO₃⁻/NO₂⁻ balance, stabilizes H₂O₂, reduces free acidity | Tailored PAW formulation, stable reactive profile |
| Plasma parameters optimization | Adjustment of discharge time, power, using different feed gases (e.g., O₂, N₂, air) | Controls RONS generation and pH | Produces crop-specific PAW chemistry; reduces over-acidification, improves antimicrobial properties and influence plants signaling |
| On-site generation and immediate use or delivery system integration | Plasma reactors and irrigation system integration | Minimizes decay of short-lived species, controls exposure time, mixing ratios and flow rates | Consistent chemistry at field application; scalable with solar energy |
| Nutrient enrichment | Macro/micronutrients supplementation | Introduces essential ions and may form reactive nutrients complexes | Boost nutrients uptake, supports growth and yield |
| Temporal control | Applying PAW at optimal post-generation intervals | Exploits RONS decay kinetics for desired chemical profile | Maximizes biological efficacy; balances safety and effectiveness |
| Synergistic additives | Combining PAW with fertilizers or bio stimulants | Improves redox and nutrients Dynamics, promotes microbial activity | Improves stress tolerance; enhances root development and soil health |
| Chelation | Add chelating agents (e.g., EDTA, citric acid) | Prevents precipitation and locks nutrients in soluble form | Maximizes micronutrient bioavailability |

Supplementary Table 4. **Overview of the studies included in meta-analysis**

| **Reference** | **Location** | **Crop name** |
| --- | --- | --- |
| Kovaliova et al., (2022) | Ukraine | Flaxseed (Linum usitatissimum) |
| Islam et al., (2019) | Bangladesh | Rapeseed (Brassica napus) |
| Seleiman et al., (2024) | Saudi Arabia | Hordeum vulgare Perilla L. (barley) |
| Fovo et al., (2024) | Cameroon | Tomato (Solanum lycopersicum L.) |
| Lukacova et al., (2021) | Slovakia | Maize (Zea mays L.) |
| Rangel-Huerta et al., (2021) | Oslo, Norway | Spinach leaves (Spinacea oleracea) |
| Aceto et al., (2024) | Italy | Tomato (Solanum lycopersicum L.) |
| Rai et al., (2022) | Bangladesh | Rice (Oryza sativa L.) |
| Mandici et al., (2022) | Romania | Triticum aestivum L. cv |
| Ruamrungsri et al., (2023) | Thailand | Green oak lettuce (Lactuca sativa L.) |
| Tonmitr and Yonesu et a., (2023) | Thailand | Radish sprouts (Raphanus sativus L.) |
| Omrani et al., (2023) | Iran | German Chamomile (Matricaria chamomilla L.) |
| Kuzin et al., (2023) | Russia | Apple |
| Lamichhane et al., (2021) | Korea | Corn plants (Zea Mays) |
| Javed et al., (2023) | Korea | Pak Choi (Brassica campestris L.) |
| Pourbagher et al., (2023) | Iran | Button mushroom |
| Guragain et al., (2021) | Nepal | Soyabean |
| Skarpa et al., (2020) | Czech Republic | Maize (Zea mays L.) |
| Kostolani et al., (2021) | Slovak Republic | Pea seeds |
| Fajdetic et al., (2022) | Croatia | Lactuca sativa L. |
| Bertaccini et al., (2015) | Italy | Tomato |
| Mohajer et al., (2024) | Iran | Cotton |
| Kostolani et al., (2021) | Slovakia | Barley and pea |
| Danilejko et al., (2021) | Russia | Sorghum bicolor, Triticum aestivum and Fragaria L |
| Jiresova et al., (2022) | Czech Republic | Wheat (Triticum aestivum L.) |
| Kuzin et al., (2023) | Russia | Apple |
| Kuzin et al., (2023) | Slovakia | Wheat (Triticum aestivum L.) |
| Hashizume et val., (2020) | Japan | Rice (Oryza sativa, cv. Aichinokaori) |
| Ndiffo Yemeli et al., (2021) | Slovakia | Maize and barley |
| Guo et al., (2023) | China | Pepper seeds |
| Guo et al., (2024) | Bangladesh | Potato (Solanum tuberosum L.) |
| Guragain et al., (2022) | Nepal | Wheat (T. aestivum) and soybean (G. max) |
| Abbaszadeh et al., (2021) | Iran | Lettuce (Lactuca sativa Linnaeus) |
| Rothwell et al., (2023) | Australia | Cucamelon |
| Vichiansan et al., (2023) | Thailand | Tomato (Solanum lycopersicum L) |
| Wang et al., (2023) | China | Wheat (Triticum aestivum L.) |
| Che et al., (2024) | China | Broccoli Seed |
| Islam et al., (2024) | Japan |  |
| Savi et al., (2024) | USA | Tomato plants (Solanum lycopersicum L.) |
| Stoleru et al., (2020) | Romania | Lettuce (Lactuca sativa Linnaeus) |
| Sajib et al., (2020) | Bangladesh | Black gram (Vigna mungo L.) |
| Rashid et al., (2021) | Bangladesh | Paddy (Oryza sativa L) |
| Than et al., (2022) | Vietnam | Lactuca sativa L |
| Rathore et al., (2022) | India | Pea (Pisum sativum L.) |
| Rashid et al., (2022) | Bangladesh | Potato (Solanum tuberosum L.) |
| Rashid and Talukder et al., (2024) | Bangladesh | Potato (Solanum tubersum L.) |
| Adhikari et al., (2019) | South Korea | Tomato |
| Groot et al., (2018) | Australia | Cotton |
| Song et al.,. (2023) | South Korea | Peanut (Arachis hypogaea L.) |
| Bansode et al., (2024) | India | Sweet orange (Citrus sinensis) |
| Chou and Ting, (2023) | Taiwan | Mung beans (Vigna radiata L.) |
| Matra et al., (2023) | Thailand | Chinese morning glory (Ipomoea aquatica) |
| Dhungana et al., (2023) | Nepal | Radish (Raphanus sativus var. longipinnatus) |
| Chalise et al., (2024) | Nepal | Chamsur, Rayo Manakamana, Rayo Marpha, and Palungo varieties |
| Guragain et al., (2021) | Nepal | Radish, fenugreek, and pea seeds |
| Date et al., (2023) | USA | Sweet Basil (O. basilicum L.) |
| Rathore and Nema et al., (2024) | India | Radish plants (Raphanus sativus L.) |
| Punith et al., (2019) | India | Tomato |
| Apasheva et al., (2019) | Russia | Cucumber |
| Hassan et al., (2021) | Bangladesh | Rice (Oryza sativa) |
| Kucerova et al., (2021) | Slovakia | lettuce (Lactuca sativa) |
| Ivankov et al., (2021) | Lithuania | Common buckwheat (Fagopyrum esculentum) |
| Ju et al., (2023) | China | Wheat |
| Porto et al., (2018) | Italy | Soybean (Glicine max) |
| Chalise et al., (2023) | Nepal | Cauliflower |
| Jiang et al., (2014) | China | Wheat (Triticum spp.) |
| Li et al., (2018) | China | Oilseed rape (Brassica napus) |
| Ran et al., (2024) | China | Lettuce |
| Rashid et al., (2021) | Bangladesh | Rice (Oryza sativa) |
| Kovalova et al., (2024) | Ukraine | Buckwheat |
| Chalise et al., (2024) | Nepal | Tejpat (Cinnamomum tamala) |
| Han et al., (2024) | Republic of Korea | Romaine lettuce |
| Chuea-uau et al., (2024) | Thailand | Rice |
| Ka et al., (2021) | Korea | Arabidopsis thaliana L. |
| Chen et al., (2023) | Taiwan | Soybean seeds [Glycine max L.) |
| Attri et al., (2023) | Japan | Radish (Raphanus sativus L.) |
| Gierczik et al., (2020) | Hungary | Barley |
| Doshi et al., (2024) | Slovakia | Wheat |
| Lee et al., (2020) | Republic of korea | Nicotiana tabacum L |
| Guragain et al, (2023) | Nepal | phapar, barley, mustard, and rayo. |
| Guragain et al., (2023) | Thailand | Cordyceps militaris (C. militaris) |
| Darmanin et al., (2021) | Malta | Mung bean |
| Chen et al., (2024) | China | Camellia (Camellia oleifera Abel.) seeds |
| Ahmad et al., (2022) | Thailand | Pepper (Capsicum annuum L.) |
| Suwannarat et al., (2024) | Thailand | Cabbage |
| Ji et al., (2022) | China | Soybean |
| Mahanata et al., (2022) | USA | Soybean (Glycine max) |
| Ali et al., (2021) | China | Tomato (Solanum lycopersicum) |
| Abouelenein et al., (2021) | Italy | Rocket-Salad (Eruca sativa Mill.) |
| Feizollahi et al., (2023) | Canada | Barley |
| Pragalalyaashree et al., (2024) | India | Button Mushroom (Agaricus bisporus) |
| Marcek et al., (2023) | Croatia | Wheat |
| Hashizume et al., (2023) | Japan | Rice (Oryza sativa L.) |
| Zuo et al., (2024) | China | Corn |
| Meng et al., (2017) | China | Wheat |
| Rashid et al., (2023) | Bangladesh | Eggplant (Solanum melangena L.) |
| Chalise et al., (2023) | Nepal | Wheat |
| Hossain et al., (2022) | Bangladesh | Maize |
| Guragain et al., (2023) | Nepal | Mustard (Brassica nigra) |
| Rivero et al., (2022) | USA | Alfalfa, broccoli and clover sprouts |
| Guo et al., (2022) | China | Wheat |
| Tan et al., (2021) | USA | Purple lettuce, kale, and baby spinach |
| Zhang et al., (2022) | China | Broccoli |
| Guo et al., (2017) | China | Grapes |
| Ndiffo Yemeli et al., (2022) | Slovakia | Pea |
| Gutiérrez-León et al., 2022 | Mexico | Lentil |
| Gutierrez-Leon et al., (2022) | Romania | Wheat (Triticum aestivum) |
| Punthi et al., (2023) | Taiwan | Pleurotus ostreatus |
| Perez et al., (2019) | Italy | Tomato |
| Than et al., (2025) | Vietnam | Mustard Greens (Brassica juncea L.) |
| Abuzairi et al., (2017) | Indonesia | water spinach (Ipomoea aqualica) |
| Zhao et al., (2021) | China | button mushrooms (Agaricus bisporus) |
| Cui et al., (2022) | China | Mung bean (Vigna radiata) |
| Thongmak et al., (2022) | Thailand | Maize |
| Waskow et al., (2022) | Switzerland | Arabidopsis thaliana |
| Tripathy and Srivastav, (2024) | India | Centella asiatica |
| Wang et al., (2022) | China | Tartary buckwheat (Fagopyrum tataricum (L.) |
| Lin et al., (2023) | China | Citrus sinensis |
| Song et al., (2024) | Republic of Korea | Soybean |
| Padureanu et al., (2018) | Romania | Lactuca Sativa L |
| Kruger and Stohr et al., (2024) | Germany | Hordeum vulgare |
| Mosovska et al., (2022) | Slovak Republic | Soybean |
| Cechova et a., (2024) | Czech republic | Cannabis sativa |
| Abbaszadeh and Boushehri, (2024) | Iran | Lettuce |
| Liu et al., (2024) | China | Kiwifruit |
| Javed et al., (2024) | Republic of Korea | Pak Choi |
| Fatelnig et al., (2024) | UK | Tef (Eragrostis tef) |
| Machado-Moreira et al., (2021) | Ireland | Alfalfa and mung bean |
| Pal et al., (2023) | India | Wheat |
| Darmanin et al., (2020) | Malta | Mung bean |
| Belov et al., (2023) | Russia | Radish |
| Hsu et al., (2023) | Taiwan | Water spinach (Ipomoea aquatica) |
| Hou et al., (2021) | Taiwan | Water spinach |
| Gao et al., (2019) | China | Pea |
| Ahn et al., (2019) | USA | Corn |
| Alves et al., (2019) | Brazil | Erythrina velutina |
| Gupta et al., (2024) | South Korea | Radish (Raphanus sativus) |
| Ma et al., (2024) | China | Agropyron Mongolicum |
| Xu et al., (2024) | China | Medicago sativa |
| Dahal et al., (2024) | Republic of Korea | Maize and Pea |
| Mansory and Bahreini, (2024) | Iran | Lentil |
| Terebun et al., (2021) | Poland | Beetroot and Carrot |
| Mandici et al., (2022) | Romania | Wheat |
| Lubin et al., (2016) | Russia | Carrot, radish and garlic |
| Sivachandiranab and Khacef et al., (2017) | France | Radish, tomato, and sweet pepper |
| Ashurov et al., (2022) | Uzbekistan | Cotton, wheat and strawberries |
| Lojkova et al., (2024) | Czech Republic | Carrot, melilot and mallow |
| Matra et al., (2022) | Thailand | Green oak lettuce |
| Pal et al., (2024) | India | Wheat |
| Bafoil et al., (2018) | France | Arabidopsis thaliana |
| Danileyko et al., (2023) | Russia | Spruce and Strawberry |
| Wang et al., (2022) | Taiwan | Lettuce Sativa |
| Konchekov et al., (2022) | Russia | Pear |
| Shaer et al., (2020) | Egypt | Wheat |
| Jiresova et al., (2021) | Czech Republic | Alfalfa (Medicago sativa L.) |
| Mohandoss et al., (2024) | India | Pearl Millet |
| Mogo et al., (2022) | Cameroon | Maize |
| Silapasert et al., (2022) | Thailand | Green microalgae (Chlorella spp.) |
| Nasibi et al., (2024) | Iran | Cut rose flowers |
| Benabderrahim et al., (2024) | Tunisia | Barley |
| Wang et al., (2023) | China | Cuimi kumquat |
| Xiao et al., (2024) | USA | Spinach |
| Zhou et al., (2016) | China | Mung bean |

# PRISMA 2020 Main Checklist

| **Topic** | **No.** | **Item** | **Location where item is reported** |
| --- | --- | --- | --- |
| **TITLE** |  |  |  |
| **Title** | 1 | Identify the report as a systematic review. | Yes |
| **ABSTRACT** |  |  |  |
| **Abstract** | 2 | See the PRISMA 2020 for Abstracts checklist |  |
| **INTRODUCTION** |  |  |  |
| **Rationale** | 3 | Describe the rationale for the review in the context of existing knowledge. |  |
| **Objectives** | 4 | Provide an explicit statement of the objective(s) or question(s) the review addresses. |  |
| **METHODS** |  |  |  |
| **Eligibility criteria** | 5 | Specify the inclusion and exclusion criteria for the review and how studies were grouped for the syntheses. |  |
| **Information sources** | 6 | Specify all databases, registers, websites, organisations, reference lists and other sources searched or consulted to identify studies. Specify the date when each source was last searched or consulted. |  |
| **Search strategy** | 7 | Present the full search strategies for all databases, registers and websites, including any filters and limits used. |  |
| **Selection process** | 8 | Specify the methods used to decide whether a study met the inclusion criteria of the review, including how many reviewers screened each record and each report retrieved, whether they worked independently, and if applicable, details of automation tools used in the process. |  |
| **Data collection process** | 9 | Specify the methods used to collect data from reports, including how many reviewers collected data from each report, whether they worked independently, any processes for obtaining or confirming data from study investigators, and if applicable, details of automation tools used in the process. | NA |
| **Data items** | 10a | List and define all outcomes for which data were sought. Specify whether all results that were compatible with each outcome domain in each study were sought (e.g. for all measures, time points, analyses), and if not, the methods used to decide which results to collect. |  |
|  | 10b | List and define all other variables for which data were sought (e.g. participant and intervention characteristics, funding sources). Describe any assumptions made about any missing or unclear information. |  |
| **Study risk of bias assessment** | 11 | Specify the methods used to assess risk of bias in the included studies, including details of the tool(s) used, how many reviewers assessed each study and whether they worked independently, and if applicable, details of automation tools used in the process. |  |
| **Effect measures** | 12 | Specify for each outcome the effect measure(s) (e.g. risk ratio, mean difference) used in the synthesis or presentation of results. |  |
| **Synthesis methods** | 13a | Describe the processes used to decide which studies were eligible for each synthesis (e.g. tabulating the study intervention characteristics and comparing against the planned groups for each synthesis (item 5)). |  |
|  | 13b | Describe any methods required to prepare the data for presentation or synthesis, such as handling of missing summary statistics, or data conversions. |  |
|  | 13c | Describe any methods used to tabulate or visually display results of individual studies and syntheses. |  |
|  | 13d | Describe any methods used to synthesize results and provide a rationale for the choice(s). If meta-analysis was performed, describe the model(s), method(s) to identify the presence and extent of statistical heterogeneity, and software package(s) used. |  |
|  | 13e | Describe any methods used to explore possible causes of heterogeneity among study results (e.g. subgroup analysis, meta-regression). |  |
|  | 13f | Describe any sensitivity analyses conducted to assess robustness of the synthesized results. |  |
| **Reporting bias assessment** | 14 | Describe any methods used to assess risk of bias due to missing results in a synthesis (arising from reporting biases). | NA |
| **Certainty assessment** | 15 | Describe any methods used to assess certainty (or confidence) in the body of evidence for an outcome. |  |
| **RESULTS** |  |  |  |
| **Study selection** | 16a | Describe the results of the search and selection process, from the number of records identified in the search to the number of studies included in the review, ideally using a flow diagram. |  |
|  | 16b | Cite studies that might appear to meet the inclusion criteria, but which were excluded, and explain why they were excluded. |  |
| **Study characteristics** | 17 | Cite each included study and present its characteristics. | S Table 2. |
| **Risk of bias in studies** | 18 | Present assessments of risk of bias for each included study. |  |
| **Results of individual studies** | 19 | For all outcomes, present, for each study: (a) summary statistics for each group (where appropriate) and (b) an effect estimate and its precision (e.g. confidence/credible interval), ideally using structured tables or plots. |  |
| **Results of syntheses** | 20a | For each synthesis, briefly summarise the characteristics and risk of bias among contributing studies. |  |
|  | 20b | Present results of all statistical syntheses conducted. If meta-analysis was done, present for each the summary estimate and its precision (e.g. confidence/credible interval) and measures of statistical heterogeneity. If comparing groups, describe the direction of the effect. |  |
|  | 20c | Present results of all investigations of possible causes of heterogeneity among study results. |  |
|  | 20d | Present results of all sensitivity analyses conducted to assess the robustness of the synthesized results. | NA |
| **Reporting biases** | 21 | Present assessments of risk of bias due to missing results (arising from reporting biases) for each synthesis assessed. | NA |
| **Certainty of evidence** | 22 | Present assessments of certainty (or confidence) in the body of evidence for each outcome assessed. |  |
| **DISCUSSION** |  |  |  |
| **Discussion** | 23a | Provide a general interpretation of the results in the context of other evidence. |  |
|  | 23b | Discuss any limitations of the evidence included in the review. |  |
|  | 23c | Discuss any limitations of the review processes used. |  |
|  | 23d | Discuss implications of the results for practice, policy, and future research. |  |
| **OTHER INFORMATION** |  |  |  |
| **Registration and protocol** | 24a | Provide registration information for the review, including register name and registration number, or state that the review was not registered. | NA |
|  | 24b | Indicate where the review protocol can be accessed, or state that a protocol was not prepared. | NA |
|  | 24c | Describe and explain any amendments to information provided at registration or in the protocol. | NA |
| **Support** | 25 | Describe sources of financial or non-financial support for the review, and the role of the funders or sponsors in the review. |  |
| **Competing interests** | 26 | Declare any competing interests of review authors. |  |
| **Availability of data, code and other materials** | 27 | Report which of the following are publicly available and where they can be found: template data collection forms; data extracted from included studies; data used for all analyses; analytic code; any other materials used in the review. |  |

#####

# PRIMSA Abstract Checklist

| **Topic** | **No.** | **Item** | **Reported?** |
| --- | --- | --- | --- |
| **TITLE** |  |  |  |
| **Title** | 1 | Identify the report as a systematic review. | Yes |
| **BACKGROUND** |  |  |  |
| **Objectives** | 2 | Provide an explicit statement of the main objective(s) or question(s) the review addresses. | Yes |
| **METHODS** |  |  |  |
| **Eligibility criteria** | 3 | Specify the inclusion and exclusion criteria for the review. | Yes |
| **Information sources** | 4 | Specify the information sources (e.g. databases, registers) used to identify studies and the date when each was last searched. | Yes |
| **Risk of bias** | 5 | Specify the methods used to assess risk of bias in the included studies. | Yes |
| **Synthesis of results** | 6 | Specify the methods used to present and synthesize results. | Yes |
| **RESULTS** |  |  |  |
| **Included studies** | 7 | Give the total number of included studies and participants and summarise relevant characteristics of studies. | Yes |
| **Synthesis of results** | 8 | Present results for main outcomes, preferably indicating the number of included studies and participants for each. If meta-analysis was done, report the summary estimate and confidence/credible interval. If comparing groups, indicate the direction of the effect (i.e. which group is favoured). | Yes |
| **DISCUSSION** |  |  |  |
| **Limitations of evidence** | 9 | Provide a brief summary of the limitations of the evidence included in the review (e.g. study risk of bias, inconsistency and imprecision). | Yes |
| **Interpretation** | 10 | Provide a general interpretation of the results and important implications. | Yes |
| **OTHER** |  |  |  |
| **Funding** | 11 | Specify the primary source of funding for the review. | Yes |
| **Registration** | 12 | Provide the register name and registration number. |  |

*From:* Page MJ, McKenzie JE, Bossuyt PM, Boutron I, Hoffmann TC, Mulrow CD, et al. The PRISMA 2020 statement: an updated guideline for reporting systematic reviews. MetaArXiv. 2020, September 14. DOI: 10.31222/osf.io/v7gm2. For more information, visit: [www.prisma-statement.org](file:///C:\Users\Nawab%20Ali\Downloads\www.prisma-statement.org)
